# Pulse transit time estimation of aortic pulse wave velocity and blood pressure using machine learning and simulated training data

**DOI:** 10.1371/journal.pcbi.1007259

**Published:** 2019-08-15

**Authors:** Janne M. J. Huttunen, Leo Kärkkäinen, Harri Lindholm

**Affiliations:** 1 Nokia Bell Laboratories, Espoo, Finland; 2 Department of Electrical Engineering and Automation, Aalto University, Espoo, Finland; University of Michigan, UNITED STATES

## Abstract

Recent developments in cardiovascular modelling allow us to simulate blood flow in an entire human body. Such model can also be used to create databases of virtual subjects, with sizes limited only by computational resources. In this work, we study if it is possible to estimate cardiovascular health indices using machine learning approaches. In particular, we carry out theoretical assessment of estimating aortic pulse wave velocity, diastolic and systolic blood pressure and stroke volume using pulse transit/arrival timings derived from photopletyshmography signals. For predictions, we train Gaussian process regression using a database of virtual subjects generated with a cardiovascular simulator. Simulated results provides theoretical assessment of accuracy for predictions of the health indices. For instance, aortic pulse wave velocity can be estimated with a high accuracy (*r* > 0.9) when photopletyshmography is measured from left carotid artery using a combination of foot-to-foot pulse transmit time and peak location derived for the predictions. Similar accuracy can be reached for diastolic blood pressure, but predictions of systolic blood pressure are less accurate (*r* > 0.75) and the stroke volume predictions are mostly contributed by heart rate.

## Author summary

Recently there has been a strong trend for self-monitoring of your cardiovascular health and new wearable sport trackers and mobile applications are coming to the market everyday. However, such solutions are mostly taking advantage of heart rate measurement. Other health indices such as blood pressure and pulse wave velocity reflecting to the condition of cardiovascular system would also be of great interest, but such solutions for continuous monitoring are barely existing or are at least unreliable. In this paper, we use computational modelling to assess theoretical capabilities of such measurements. We concentrate on predicting health indices using on pulse transmit time type of measurements. Such measurements could be carried out, for example, with photopletyshmography sensor or an optical sensor already found from several wearable sport trackers. We use cardiovascular modelling to create a database of “virtual subjects”, which is applied with machine learning to construct predictors for health indices. Our findings suggest that aortic pulse wave velocity and diastolic blood pressured could be predicted with a high accuracy, but predictions of systolic blood pressure are less accurate.

## Introduction

This paper considers continuous monitoring of cardiac health using computational modelling. Stiffening of the arterial wall, such as aorta, causes reduction in the pulsatile properties in the vascular tree, accelerates the vascular premature ageing and predisposes to the dysfunction of the heart, brain and other organs [1, 2]. Aortic stiffness can be measured by using invasive methods or medical imaging such as ultrasound [3] and MRI [2]. Another indicator reflecting the cardiac performance is stroke volume (SV), which is typically measured using Doppler ultrasound [4]. However, these imaging modalities typically require special expertise and are only carried out clinically. On the other hand, aortic stiffness is associated with the unfavourable changes in the diastolic and systolic blood pressures (DBP/SBP), which can have several negative consequences in cardiac function and structure [1]. Ambulatory home measurements of DBP and SBP use the techniques based on inflated cuffs, but continuous recording is still cumbersome. It would be helpful to find unobtrusive methods for the long-term monitoring of these cardiac indices during the daily activities and sleep.

Arterial stiffness is often assessed by measuring pulse wave velocity (PWV), which is increased in stiffer arteries. The PWV can be estimated by measuring arrivals of pulse waves at two arterial sites:

$$\text{PWV}=\frac{\text{distance}\;\text{between}\;\text{the}\;\text{sites}}{\text{travel}\;\text{time}\;\text{between}\;\text{the}\;\text{sites}}.$$

The travel time is commonly referred as pulse transit time (PTT). Arrival of the pulse wave to distal arterial sites can be easily measured by using a photoplethysmogram (PPG), which is an optical non-invasive sensor that can be placed, for example, in a wearable device [5]. On the other hand, in order to predict aortic stiffness reliably, the first arterial site should be located at the beginning of aorta (for measurement of aortic valve opening). However, a measurement of valve opening can require a device such as phonocardiograph, ultrasound or MRI.

To overcome this difficulty, PTT is often approximated using pulse arrival time (PAT) which uses the R- wave of electrocardiogram (ECG) as a reference timing [6]. However, there exists controversy in the clinical accuracy of using PAT in the predictions due to variations in pre-ejection period (PEP) from the R-wave to aortic valve opening [7, 8]. An alternative approach is to approximate the reference with a measurement from another distal site near aorta. For example, the gold standard for aortic PWV measurement is to measure differences of pulse arrivals to carotid and femoral arteries.

The estimation of blood pressure from arrival of pulse waves has also been largely studied; see e.g. [6, 9, 10]. Although promising results have been reported, clinical use of these techniques is still limited. Haemodynamic alterations can have significant effects on the accuracy [11].

A common problem with the clinical use of the above methodologies is that the development and validation of the methods typically require a large set of measurements from real human subjects with sufficient variety. Such data collection can be a very difficult and expensive task.

A preliminary assessment of the methods without extensive data collection can be carried out using simulators. For example, Willemet et al [12, 13] proposed approach to use cardiovascular simulator for generation of a database of “virtual subjects” with sizes limited only by computational resources. In their study, the databases were generated using one-dimensional (1D) model of wave propagation in a artery network comprising of largest human arteries [14]. Such 1D models provide computationally efficient way to simulate blood circulation and are also used in several other applications [15]. There are also studies validating 1D simulations against real measurement [16–18]. The virtual database approach was used to assess accuracy of pulse wave velocity measurements for estimation of aortic stiffness [12] and the accuracy of pulse wave analysis algorithms [13].

The aim of our study is to assess theoretical limitations for the prediction of aortic pulse wave velocity (aPWV), blood pressures (DBP/SBP) and SV from PTT/PAT measurements. We apply a similar virtual database approach to find correlations between these cardiac indices and PTT/PAT timings measured from different locations. In particular, we train Gaussian process regressor to predict the cardiac indices using different combinations of PTT and PAT measurements. The regressor model is trained using a large set of virtual subjects generated using 1D cardiovascular simulator, and the results are validated using another set of virtual subjects. The result of study can give preliminary implications for the accuracy of such predictions in rather ideal circumstances.

Our study is based on the 1D haemodynamic model of entire adult circulations introduced by Mynard and Smolich [19]. It includes heart functions and all larger arteries and veins for both systemic and pulmonary circulation. As heart is included to the model, it can also simulate variations in PEP that are essential in the comparison of PTT and PAT timings.

This paper is organized as follows. Cardiovascular model, generation of virtual subjects and prediction methods are described in Methods and Models section. Results section contains numerical experiments. We will finish with Discussion.

## Methods and models

In this section, we begin with a short summary of cardiovascular model and present its numerical discretization.We will also describe the generation of the database of virtual subjects and the computation of Gaussian process predictions.

### Blood circulation model

The blood circulation model is based on the 1D haemodynamic model described in [19], which basically extends commonly used 1D wave dynamics model (see e.g. [14]) with heart functions and realistic arteria and venous networks including pulmonary and coronary circulations. The components of the model are shortly summarized below, see [19] for more details.

#### One-dimensional wave dynamics

**Fig 1:**
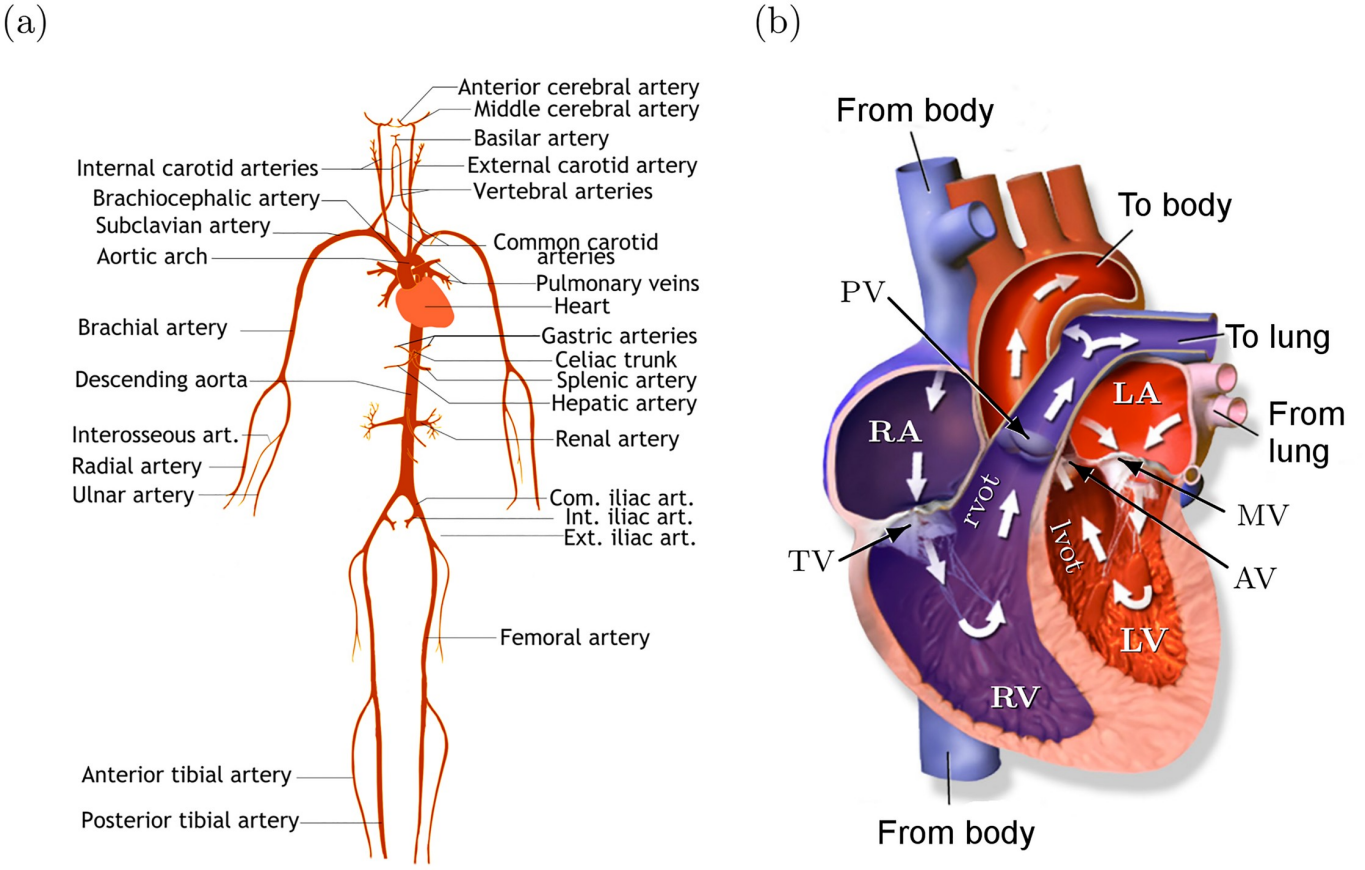
(a) Illustration of human arterial system. The picture includes only a few largest arteries; see [19] for the complete set of arteries and veins used in the model. (b) Illustration of human heart including four chambers: left atrium (LA), left ventricle (LV), right atrium (RA) and right ventricle (RV). Left and right ventricular outflow track (lvot/rvot) are short 1D segments before the valves. Valves: tricuspid valve (TV), pulmonary valve (PV), mitral valve (MV) and aortic valve (AV). Picture by BruceBlaus (CC BY).

Human arterial network is illustrated in Fig 1(a). In 1D modelling, the arterial system is divided into segments (e.g. from aortic root to the branching point of brachiochephalic artery; see e.g. [14, 19]). Each segment is assumed to be a straight compliant tube with the length *L*. The circular cross-sectional area *A*(*x*, *t*) and the velocity profile *U*(*x*, *t*) are assumed to depend on time *t* and a single axial coordinate *x* ∈ [0, *L*]. To radial direction, the velocity profile is assumed to be axisymmetric and flat which agrees relatively well to experimental data (see e.g. [16]). The governing (nonlinear) equations can be written as [14, 19],

$$\frac{\partial A}{\partial t}+\frac{\partial AU}{\partial x}=0,$$ (1)

$$\frac{\partial U}{\partial t}+U\frac{\partial U}{\partial x}+\frac{1}{\rho }\frac{\partial p}{\partial x}=\frac{f}{\rho A},$$ (2)

where *p* is the pressure, *ρ* and *μ* are the density and viscosity of blood, and *f* is the frictional force. With the axisymmetric and flat velocity profile, the frictional force can be written as *f* = −22*μπU* [14].

The pressure-area relationship is written as [19, 20]

$$\begin{array}{c} p=p\left(A\right)=P_{0}+\frac{2\rho c_{0}^{2}}{b}\left[{\left(\frac{A}{A_{0}}\right)}^{b/2}-1\right], \end{array}$$ (3)

where *A*_0_, *P*_0_ and *c*_0_ are the cross-sectional area, the pressure and the wave speed at a reference state. We have omitted the wall-viscosity in this study since the treatment of the viscosity would result in significantly higher demands in numerical discretization (remind that our aim is to run the model repeatedly). We choose *b* = 1 which corresponding to the pressure law used in Alastruey’s model [14, 16]. In Mynard et al [19, 20], the constant *b* was specified as $b=2\rho c_{0}^{2}/\left(P_{0}-P_{\text{collapse}}\right)$ where *P*_collapse_ is the collapse pressure. However, in our experiments, this choice led to very steep raises in pressures during systolic period due to omitted viscosity.

#### Heart and valves

**Fig 2:**
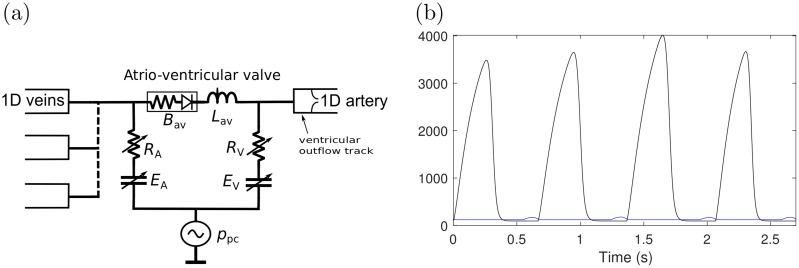
(a) Schematic of atrioventriclular (av) model. *B* is the Bernoulli valve resistance, *R* is the source resistance, *L* is the blood inertance and *E* is the elastance of the wall. The subscripts A and V refer to atrial and ventricular, respectively, and *p*_pc_ is the pericardiac pressure. (b) Freewall elastance *E*_fw_ for LA (blue) and LV (black). The figure includes four pulses. The duration of the pulse, the maximum elastance *E*_max_ and timing parameters *τ*_1_ and *τ*_2_ vary between pulses.

The anatomy of heart and blood circulation through heart are illustrated in Fig 1(b). The blood flow through atriums (LA/RA) and ventricles (LV/RV) is modelled using a lumped parameter model introduced in [20], which was extended to include interactions between heart chambers and pericardiac pressure in [19]. The model is illustrated in Fig 2(a).

The relationship between the flow through valves (*q*) and the transvalvular pressure difference Δ*p* (= *p*_in_ − *p*_out_) is given by the Bernoulli equation,

$$\begin{array}{c} \Delta p=B_{\text{av}}q\left|q\right|+L_{\text{av}}\frac{dq}{dt}, \end{array}$$ (4)

where the Bernoulli resistance *B*_av_ and the blood inertance *L*_av_ are

$$\begin{array}{c} B_{\text{av}}=\frac{\rho }{2A_{\text{eff}}^{2}}\;\text{and}\;L_{\text{av}}=\frac{\rho l_{\text{eff}}}{A_{\text{eff}}}, \end{array}$$ (5)

where *A*_eff_ and *l*_eff_ are the effective valve orifice area and length. The valve dynamics are modelled using a state variable *ξ* which represents the state of the valve (0 ≤ *ξ* ≤ 1, *ξ* = 0 for closed, *ξ* = 1 for open) such that *A*_eff_(*t*) = (*A*_eff,max_ − *A*_eff,min_)*ξ*(*t*) + *A*_eff,min_. Valve dynamics are modelled by

$$\begin{array}{c} \frac{d\xi }{dt}=K_{\text{vo}}\left(1-\xi \right)\Delta p\text{when}\Delta p\geq 0\;\text{or}\;\frac{d\xi }{dt}=K_{\text{vc}}\xi \Delta p\text{when}\Delta p<0, \end{array}$$ (6)

where *K*_vo_ and *K*_vc_ are rate coefficients for the valve opening and closing, respectively.

The relationship between the pressure *p* and the volume *V* of a heart chamber is given by

$$\begin{array}{c} p=p_{\text{pc}}+\frac{E_{\text{nat}}}{E_{\text{sep}}}p^{*}+E_{\text{nat}}\left(V-V_{p=0}\right)-R_{s}q, \end{array}$$ (7)

where *p*_pc_ is the pericardiac pressure (assumed to depend exponentially on the total chamber volumes; see [19]), *E*_nat_ is the native elastance of the chamber, *E*_sep_ is the septal elastance, *V*_*p* = 0_ is the volume of the chamber in zero pressure, *R*_s_ is the source resistance, and p* is the pressure in the contralateral chamber. The native elastance of a chamber is given by

$$\begin{array}{c} E_{\text{nat}}=\frac{E_{\text{fw}}E_{\text{sep}}}{E_{\text{fw}}+E_{\text{sep}}}-\mu_{\text{AV}}q, \end{array}$$ (8)

where *E*_fw_ is the freewall elastance of the chamber and *μ* is the atrioventricular plane piston constant. The time varying freewall elastances for each chamber are modelled by

$$\begin{array}{c} E_{\text{fw}}=k\left(\frac{g_{1}}{1+g_{1}}\right)\left(\frac{1}{1+g_{2}}\right)+E_{\text{fw}}^{\text{min}},\;\text{where}\;g_{i}={\left(\frac{t-t_{\text{onset}}}{\tau_{i}}\right)}^{m_{i}},\;i=1,2, \end{array}$$ (9)

and *k* is the scaling constant chosen such that $\max\left(E_{\text{fw}}\right)=E_{\text{fw}}^{\text{max}}$. The functional properties of heart are specified via the maximum and minimum free wall elastances ($E_{\text{fw}}^{\text{min}/\text{max}}$), the timing parameters *τ*_1_, *τ*_2_ and *t*_onset_ and the slope parameters *m*_1_ and *m*_2_. For example, increasing $E_{\text{fw}}^{\text{max}}$ increases the contraction of the heart and the length of the pulse can be adjusted through *τ*_1_ and *τ*_2_. Fig 2(b) shows an example of the form of *E*_fw_.

#### Vascular beds

**Fig 3:**
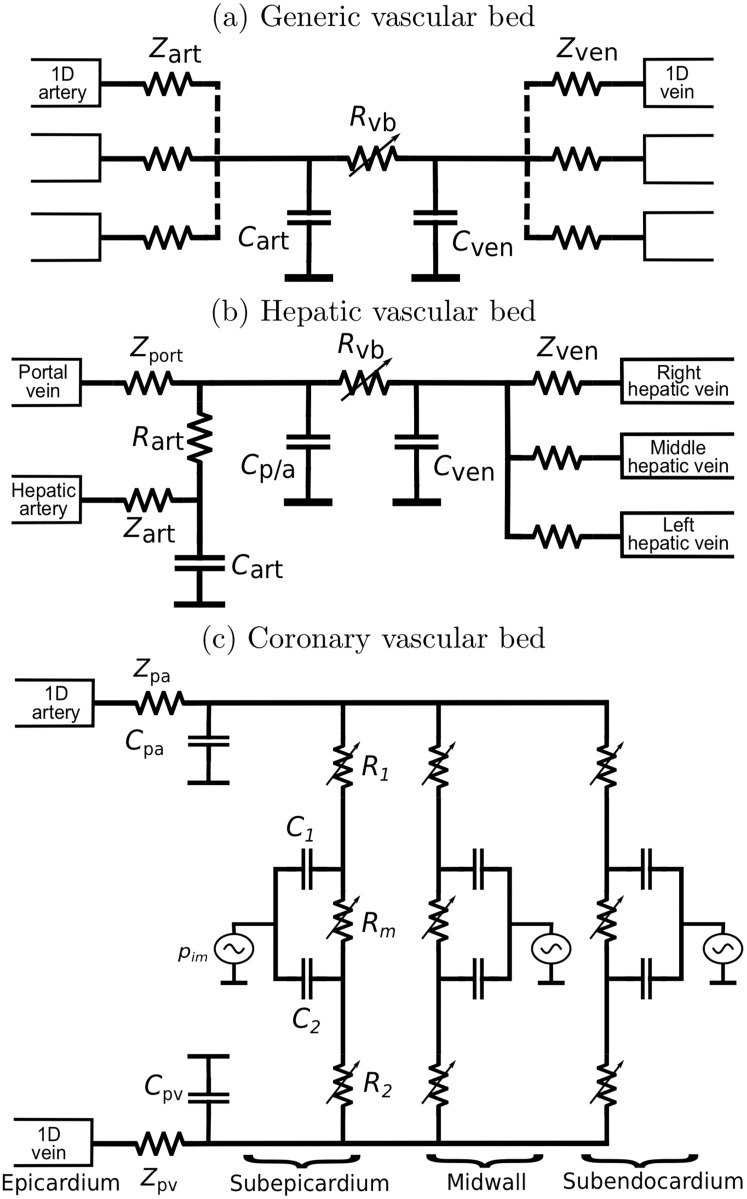
(a) Generic vascular bed model; (b) Hepatic vascular bed model with arterial and venous inlets; (c) Coronary vascular bed model with compartments representing subepicardial, midwall and subendocardial layers.

Mynard and Smolich [19] describe models for circulation through three types of vascular beds (Fig 3): generic vascular beds, a hepatic vascular bed and coronary vascular beds. The generic vascular bed model (Fig 3(a)) is used for all microvasculature beds except the liver and myocardium. It is based on commonly used three-element Windkessel model and consists of the characteristic impedances *Z*_art_ and *Z*_ven_(to couple the connecting 1D arteries to the vascular bed), lumped compliances for the arterial and venous microvasculature (*C*_art_ and *C*_ven_) and the vascular bed resistance *R*_vb_. The resistance is assumed to be pressure dependent to account for the fact that the atriovenous pressure difference remains positive even with zero vascular bed flow:

$$\begin{array}{c} R_{\text{vb}}=\{\begin{array}{cc} R_{0}\left(\frac{p_{\text{tm0}}-P_{\text{zf}}}{p_{\text{tm}}-P_{\text{zf}}}\right), & p_{\text{tm}}>P_{\text{zf}},\\ \infty , & p_{\text{tm}}\leq P_{\text{zf}}, \end{array} \end{array}$$ (10)

where *p*_tm_ = *p* − *p*_ext_ is the transmural pressure, *P*_zf_ is the zero-flow pressure and *R*_0_ is the reference resistance.

The hepatic vascular bed (Fig 3(b)) is a modification of the above to account for both arterial and venous inlets in liver. It includes a compartment for the flow from hepatic artery (*R*_art_, *C*_art_) which connects to another compartment (*C*_p/a_) with common portal/arterial pressure.

The coronary vascular bed model (Fig 3) represents blood flow through intramyocardial. The coronary vessels experience a large time-varying myocardial pressures *p*_im_ caused by the contracting heart muscle. To model depth-wise myocardial pressure, the model includes three layers representing subendocardium, midwall and subepicardium, each layer having three non-linear resistances *R*_1_, *R*_*m*_ and *R*_2_:

$$\begin{array}{c} R_{i}\left(t\right)=R_{0,i}\frac{V_{0,i}^{2}}{V_{i}^{2}},\;\;i=1,2,\;R_{m}\left(t\right)=R_{0,m}(\frac{V_{0,1}^{2}}{V_{1}^{2}}+\frac{V_{0,2}^{2}}{V_{2}^{2}}), \end{array}$$ (11)

where the blood volumes *V*_1_ and *V*_2_ are are given by

$$\begin{array}{c} V_{i}\left(t\right)=V_{0,i}+\int_{0}^{t}C_{i}\frac{dp_{\text{tm},i}\left(t^{\prime }\right)}{dt}dt^{\prime },\;p_{\text{tm},i}=p-p_{\text{im}},\;i=1,2. \end{array}$$ (12)

The intramyocardial pressures *p*_im_ is assumed to be the sum of pressure transmitted from the ventricular cavity into the heart muscle and pressure generated mechanically by the thickening heart muscle. See [19] for details.

#### Numerical solution of the cardiovascular model

Our numerical solution of the wave propagation model is based on the discontinuous Galerkin (DG) method. The derivation of the DG solution for the 1D wave model (1)–(2) is described with details e.g. in [14], and therefore it is only briefly summarized here. We will give more details about the treatment of heart chambers, valves and vascular beds as the numerical treatment differs from [19] due to the different numerical scheme.

The Eqs (1)–(2) can be written in a conservative form as [14]

$$\begin{array}{c} \frac{\partial U}{\partial t}+\frac{\partial F}{\partial x}=\hat{S},\;U=\left(\begin{array}{c} A\\ U \end{array}\right),\;F=\left(\begin{array}{c} AU\\ \frac{U^{2}}{2}+\frac{p}{\rho } \end{array}\right)\;\;\text{and}\;\hat{S}=\left(\begin{array}{c} 0\\ \frac{f}{\rho A} \end{array}\right), \end{array}$$ (13)

where ***F*** is called the flux term. As in the standard finite element method (FEM), each (arterial or venous) 1D segment [0, *L*] is divided into non-overlapping elements *Ω*_*e*_. In addition, (13) is multiplied with a (vector valued) test function ***ψ*** and integrated over the segment. Then the integration by parts gives

$$\overset{N_{\text{el}}}{\underset{e=1}{\sum^{\text{​}}}}\left[{\left(\frac{\partial U}{\partial t},\psi \right)}_{\Omega_{e}}-{\left(F,\frac{\partial \psi }{\partial x}\right)}_{\Omega_{e}}+{\left[F\cdot \psi \right]}_{x_{e}^{l}}^{x_{e}^{r}}\right]=\overset{N_{\text{el}}}{\underset{e=1}{\sum^{\text{​}}}}{\left(\hat{S},\psi \right)}_{\Omega_{e}},$$

where (***u***, ***v***)_*Ω*_ = ∫_*Ω*_ ***u*** ⋅ ***v** dx* is the standard *L*^2^(*Ω*) inner product. For a numerical solution, ***U*** and ***ψ*** are approximated which piecewise polynomial vector functions ***U***^*δ*^ and ***ψ***^*δ*^. However, contrary to the standard FEM, the approximation ***U***^*δ*^ is not enforced to be continuous across the element boundaries. Another application of the integration by parts gives

$$\begin{array}{l} \begin{array}{l} \sum_{e=1}^{N_{\text{el}}}\left[{\left(\frac{\partial U^{\delta }}{\partial t},\psi^{\delta }\right)}_{\Omega_{e}}+{\left(\frac{\partial F\left(U^{\delta }\right)}{\partial x},\psi^{\delta }\right)}_{\Omega_{e}}+{\left[\psi^{\delta }\cdot \left(F^{*}-F\left(U^{\delta }\right)\right)\right]}_{x_{e}^{l}}^{x_{e}^{r}}\right]\\ =\sum_{e=1}^{N_{\text{el}}}{\left(\hat{S}\left(U^{\delta }\right),\psi^{\delta }\right)}_{\Omega_{e}}, \end{array} \end{array}$$ (14)

where the term F* is the (approximative) flux function (determined below). The flux F* is responsible of propagating information through the elements interfaces and is also the key element in the specification of the boundary conditions for the 1D blood vessel segments.

In order to apply a numerical integration scheme for temporal discretization, we need to find F such that $\frac{\partial U^{\delta }}{\partial t}=F\left(U^{\delta }\right)$. As in the standard FEM, this corresponds to finding the coefficients of the approximation of $\frac{\partial U^{\delta }}{\partial t}$ such that (14) is satisfied for a chosen set of test functions. However, since the approximation is discontinuous in DG, the coefficients can be solved separately for each element. The problem is further simplified by using Legendre polynomials as the basis functions of the approximation and test functions, which allows us to treat each basis function separately due to *L*^2^-orthogonality.

For the numerical integration, the second-order Adams-Bashforth time integration scheme is used; see [14] for more details.

#### Characteristic analysis and the flux F*

The determination of the flux F* and numerical boundary conditions is based on the Riemann’s method of characteristics. The characteristic functions (or Riemann’s variables) of the system (13) can be written as (see [14] for the derivation)

$$\begin{array}{c} W_{f}\left(A,U\right)=U-U_{0}+\int_{A_{0}}^{A}\frac{c}{A}\;dA,\;W_{b}\left(A,U\right)=U-U_{0}-\int_{A_{0}}^{A}\frac{c}{A}\;dA, \end{array}$$ (15)

where the subscripts *f* and *b* refer to information moving to forward and backward directions, respectively, and *c* is the wave speed (local PWV),

$$\begin{array}{c} c=\sqrt{\frac{A}{\rho }\frac{\partial p}{\partial A}}. \end{array}$$ (16)

When considering the pressure-area relationship (3),

$$\begin{array}{c} c=c_{0}{\left(\frac{A}{A_{0}}\right)}^{b/4}\;\text{and}\;\int_{A_{0}}^{A}\frac{c}{A}dA=\frac{4c_{0}}{b}\left[{\left(\frac{A}{A_{0}}\right)}^{b/4}-1\right]=:\psi \left(A\right). \end{array}$$ (17)

The fluxes F* at the interfaces of elements are calculated as a solution of a Riemann problem with suitable boundary conditions, see e.g. [14]. The procedure involves finding a unique state (A*, U*) such that

$$\begin{array}{c} W_{f}\left(A_{L},U_{L}\right)=W_{f}\left(A^{*},U^{*}\right)\;\text{and}\;W_{b}\left(A_{R},U_{R}\right)=W_{f}\left(A^{*},U^{*}\right), \end{array}$$ (18)

where the subscript *L* and *R* refer to the value of *A* and *U* on the left or the right side of the boundary of the element, respectively. The flux is then given as F* = ***F***(A*, U*).

The boundary conditions for the 1D blood vessel segments are handled similarly by finding a state (A*, U*) satisfying conditions similar to (18). Treatment of the boundary conditions related to splitting and merging arteries/veins is presented in [14]. Treatment of the boundary conditions related to the heart, valve and vascular beds is presented below.

#### Numerical model for heart chambers

We consider left heart (right heart is handled similarly). The Trapezoidal rule applied to the net flow arriving to LV gives (see Fig 1(b))

$$\begin{array}{c} q_{\text{LV}}=-\frac{dV_{\text{LV}}}{dt}=q_{\text{lvot},\text{in}}-q_{\text{MV}}\;\Rightarrow \;V_{\text{LV}}^{n}=V_{\text{LV}}^{n-1}-\frac{\Delta t}{2}(q_{\text{LV}}^{n}+q_{\text{LV}}^{n-1}), \end{array}$$ (19)

where the superscript *n* refers to the *n* ’th temporal discretization point and Δ*t* is the time step. The above equation can be substituted to (7) to give

$$\begin{array}{c} p_{\text{LV}}^{n}=p_{\text{ext}}^{n}+E_{\text{nat}}^{n}[V_{\text{LV}}^{n-1}-\frac{\Delta t}{2}(q_{\text{LV}}^{n}+q_{\text{LV}}^{n-1})-V_{p=0,\text{LV}}]\left(1-K_{s,\text{LV}}q_{\text{LV}}^{n}\right), \end{array}$$ (20)

where $p_{\text{ext}}^{n}=p_{\text{LV},\text{pc}}^{n}+E_{\text{nat}}^{n}/E_{\text{sep}}^{n}p_{\text{RV}}^{n}$.

The output of LV is connected to the inlet of lvot-segment; see Fig 1(b). At the inlet of lvot, we have

$$\begin{array}{c} W_{b}\left(A_{\text{in}}^{\text{lvot}},U_{\text{in}}^{\text{lvot}}\right)=W_{b}\left(A^{*},U^{*}\right)=U^{*}-\psi \left(A^{*}\right), \end{array}$$ (21)

where $A_{\text{in}}^{\text{lvot}}$ and $U_{\text{in}}^{\text{lvot}}$ are the DG approximations at the inlet. Since $q_{\text{lvot},\text{in}}^{n}=A^{*}U^{*}$,

$$\begin{array}{c} q_{\text{LV}}^{n}=A^{*}U^{*}-q_{\text{MV}}^{n}=A^{u}\left({\tilde{W}}_{b}+\psi \left(A^{*}\right)\right)-q_{\text{MV}}^{n}=A^{*}{\tilde{W}}_{b}-q_{\text{MV}}^{n}+A^{*}\psi \left(A^{*}\right), \end{array}$$ (22)

where ${\tilde{W}}_{b}=W_{b}\left(A_{\text{in}}^{\text{lvot}},U_{\text{in}}^{\text{lvot}}\right)$. Plugging in (22) and $p_{\text{LV}}^{n}=p_{\text{lvot},\text{in}}^{n}=p\left(A^{*}\right)$ to (20) gives an equation from which A* can be solved using Newton’s method. Finally, U* can be solved from (21) and $V_{\text{LV}}^{n}$ and $p_{\text{LV}}^{n}$ are obtained during the iteration.

The atriums have multiple vein connections; see Fig 1(b). Let $A_{\text{out}}^{j}$ and $U_{\text{out}}^{j}$ be the DG approximations at the outlet of the *j*’th connecting 1D-segment (*j* = 1, …, *J*). We can write $W_{f}\left(A_{\text{out}}^{j},U_{\text{out}}^{j}\right)=W_{f}(A_{j}^{*},U_{j}^{*})=U_{j}^{*}+\psi \left(A_{j}^{*}\right)$, and further

$$\begin{array}{c} q_{\text{LA}}^{n}=q_{\text{MV}}^{n}-\sum_{j}A_{j}^{*}U_{j}^{*}=q_{\text{MV}}^{n}-\sum_{j}A_{j}^{*}({\tilde{W}}_{f}^{j}-\psi_{j}\left(A_{j}^{u}\right)). \end{array}$$ (23)

where ${\tilde{W}}_{f}^{j}=W_{f}\left(A_{\text{out}}^{j},U_{\text{out}}^{j}\right)$ and *ψ*_*j*_ is the function (17) with the parameters *A*_0_ and *c*_0_ corresponding to the outlet of *j*’th segment. Then similarly as above, we can obtain a group of *J* equations from which $A_{1}^{*},\ldots ,A_{J}^{*}$ can be simultaneously solved using Newton’s method. However, the multi-dimensional problem can be avoided by noticing that the pressure-area relationship (3) can be inverted easily (i.e. we can find *A* = *A*(*p*)). Then it is equivalent to solve *p* from the one-dimensional problem

$$\begin{array}{c} p=p_{\text{ext},\text{LA}}^{n}+E_{\text{nat}}^{n}\left[V_{\text{LA}}^{n-1}-\frac{\Delta t}{2}\left(q_{\text{LA}}^{n}\left(p\right)+q_{\text{LA}}^{n-1}\right)-V_{p=0,\text{LA}}\right]\left(1-K_{\text{s},LA}{\tilde{q}}_{\text{LA}}^{n}\left(p\right)\right). \end{array}$$ (24)

where ${\tilde{q}}_{\text{LA}}^{n}\left(p\right)$ is given by (23) with $A_{j}^{*}=A_{j}\left(p\right)$, where the subscript *j* refers to the mapping in which the parameters *A*_0_, *c*_0_ and *b* in (3) are specified for at the outlet of the *j*’th segment.

#### Valves

The application of the forward Euler method to (4) gives

$$\begin{array}{ccc} & & q^{n+1}=q^{n}+\frac{\Delta t}{L_{\text{av}}}(\Delta p^{n}-B_{\text{av}}q^{n}\left|q^{n}\right|). \end{array}$$ (25)

The Eq (6) is discretized similarly. For MV and TV, the transvalvular pressure is the pressure difference between artium and ventricle (e.g. $\Delta p^{n}=p_{\text{LA}}^{n}-p_{\text{LV}}^{n}$ for MV).

PV and AV are between 1D segments (e.g. AV is between lvot and the first segment of aorta, see Fig 1(b)). For the outlet of the ventricular outflow tracks, we specify the outflow condition (e.g. $q_{\text{lvot}}^{\text{out}}=q_{\text{AV}}^{n}$). For the inlet of the 1D segments behind the valve, we specify the inflow to be $q_{\text{valve}}^{n}$. These inflow and outflow boundary conditions can be treated similarly as above by finding the states (A*, U*); see e.g. [14] for details. Then the pressures on the both sides of the valve can be computed using the states A* and the pressure-area relationship (3).

#### Vascular beds

We consider the generic vascular bed model (Fig 3(a)). Arterial and venous flows *q*_art_ and *q*_ven_ in the generic vascular bed model (sums of all flows from/to 1D-segments) are given by

$$\begin{array}{c} q_{\text{art}}=q_{\text{cap}}+C_{\text{art}}\frac{dp_{\text{art}}}{dt},\;q_{\text{ven}}=q_{\text{cap}}-C_{\text{ven}}\frac{dp_{\text{ven}}}{dt}. \end{array}$$ (26)

The forward Euler method gives

$$\begin{array}{c} p_{\text{art}}^{n+1}=p_{\text{art}}^{n}+\frac{\Delta t}{C_{\text{art}}}\left(q_{\text{art}}^{n}-q_{\text{cap}}^{n}\right),\;p_{\text{art}}^{n+1}=p_{\text{art}}^{n}+\frac{\Delta t}{C_{\text{ven}}}\left(q_{\text{cap}}^{n}-q_{\text{ven}}^{n}\right). \end{array}$$ (27)

The capillary flows $q_{\text{cap}}^{n}$(flow through *R*_vb_) are calculated using Ohm’s law.

Vascular beds are connected to the 1D model as the terminal resistance boundary condition similarly as in [14]. For example, we consider coupling of a 1D-arterial segment to the generic vascular bed model (Fig 3(a)). The flow *q* though impedance *Z*_art_ is given by Ohm’s law $Z_{\text{art}}q=p_{1D}-p_{art}^{n}$. We need to find (A*, U*) such that

$$\begin{array}{c} Z_{\text{art}}A^{*}U^{*}=p\left(A^{*}\right)-p_{art}^{n}\;\text{and}\;W_{f}\left(A_{L},U_{L}\right)=W_{f}\left(A^{*},U^{*}\right)=U^{*}+\psi \left(A^{*}\right) \end{array}$$ (28)

The states A* and U* can be solved by combining the equations as above and applying Newton’s method. Then $q_{\text{art}}^{n}$ is the sum of flows from all 1D-outlets (A*U*).

Portal and coronary models in Fig 3(b) and 3(c) can be treated similarly.

### Virtual database

The database is created by running the cardiovascular model repeatedly. The model parameters are varied to reflect variations between individual (virtual) subjects.

In [12, 13], the seven parameters were varied: elastic artery PWV, muscular artery PWV, the diameter of elastic arteries, the diameter of muscular arteries, heart rate (HR), SV and peripheral vascular resistance. In their study, the parameters were varied by specifying a few possible values for each parameter and the cardiovascular model was run for all of the resulting 7776 combinations. However, in our study, the cardiovascular model has significantly more model parameters (e.g. parameters related to heart model and valves, vascular beds, …). Such systematic variation of all essential parameters would lead to excessively large number of combinations.

In this study, we choose “sampling” approach in which the model parameters are varied randomly. Our aim is to choose random variations that would represent healthy subject and, where applicable, the range of the parameters is of similar range as in [12]. Some choices can be rather subjective due to the limited amount of (probabilistic) information from related physiological quantities. Our goal is to choose variations to be wide enough so that “real world” can be considered as a subset of the population covered by the variations. However, if more sufficient information about parameters becomes available, it should be rather straightforward to carry out the analysis with the adjusted distributions.

In the following, the superscript (*s*) refers to a virtual subject for which the parameters are specified. The overbar notation (e.g. $\bar{L}$) refers to the values used in [19] (the baseline). Unless otherwise mentioned, the variations are chosen to be normally distributed. Furthermore, the statements such as 10% relative variation should be understood in terms of standard deviations instead explicit ranges of the parameter. We use slightly unconventional notation N(μ,X%) to denote the Gaussian distribution with mean *μ* and the standard deviation *σ* = *X*/100*μ* (i.e. *X*% variation relative to the mean/baseline). The uniform distribution is denoted as U(a,b).

#### Vascular networks

The arterial and venous network structure is chosen to be same as in [19] (the length *L* and *A*_0_ at the inlet and outlet for each 1D segment are given in their supplementary materials). To include individual variations of subjects, the lengths are chosen as

$$\begin{array}{c} L_{\ell }^{\left(s\right)}={\bar{L}}_{\ell }a^{\left(s\right)}b_{\ell }^{\left(s\right)},\;a^{\left(s\right)}∼N\left(1,10\%\right),\;b_{\ell }^{\left(s\right)}∼N\left(1,2\%\right), \end{array}$$ (29)

where the subscript *ℓ* refers to the *ℓ*th segment. The multiplier *a*^(*s*)^ can be understood as a variation in the height of subject total length and $b_{\ell }^{\left(s\right)}$ represents individual variations of blood vessel segments. With these choices, for example, distances from aortic root to the measurement locations (see below) are 17.0 ± 1.8 cm (left carotid artery) and 88.9 ± 9.1 cm (femoral artery) which are similar to the distances reported in [21, 22]. The arterial diameters *D*_0_($\propto \sqrt{A_{0}}$) are also varied similarly, except we use separate common multipliers *a*^(*s*)^ for aorta (20% variation) and rest of segments (10% variation).

The elasticity *E* of blood vessels is controlled by the reference wave speed (PWV), which can be expressed using the empirical formula [23]

$$\begin{array}{c} c_{0}^{2}=\frac{2}{3\rho }\frac{Eh}{2r_{0}}=\frac{2}{3\rho }[k_{1}\exp\left(k_{2}r_{0}\right)+k_{3}], \end{array}$$ (30)

where *r*_0_ is the reference radius, *h* is the thickness of the wall and *k*_1_, *k*_2_ and *k*_3_ are empirical constants. Elasticity of systemic arteries, especially aorta, have largest effect to the condition of the cardiovascular system (increased significantly during ageing). Therefore, aorta and other systemic arteries are chosen to include largest variations:

$$\begin{array}{cccc} & k_{1,3}^{\left(s\right)}={\bar{k}}_{1,3}{\left(\alpha_{a}^{\left(s\right)}\beta^{\left(s\right)}\right)}^{2}, & & (\text{aorta}),\\ & k_{1,3}^{\left(s\right)}={\bar{k}}_{1,3}{\left(\alpha_{a}^{\left(s\right)}\right)}^{2}, & & (\text{other}\;\text{systemic}\;\text{arteries}),\\ & k_{1,3}^{\left(s\right)}={\bar{k}}_{1,3}{\left(\gamma^{\left(s\right)}\right)}^{2}, & & (\text{all}\;\text{other}\;\text{blood}\;\text{vessels}), \end{array}$$

where $\alpha^{\left(s\right)}∼N\left(1,25\%\right)$, $\beta^{\left(s\right)}∼U\left(1,2.5\right)$ and $\gamma^{\left(s\right)}∼N\left(1,10\%\right)$. The coefficient *α*^(*s*)^ produces 25% variation to the PWV of systemic arteries which, for aorta, is further amplified with *β*^(*s*)^ giving 65% maximum variation. The slope *k*_2_ is also varied with 5% variation. To produce small variation between segments, additional 1% variation is added to the local PWV (*c*_0_) of each segment.

#### Heart functions and valve model parameter

The duration of the pulses *T*_*c*_ are chosen as follows. For each subject, HR is drawn from $N(75\;{\text{min}}^{-1},35\%)$, which is rejected if HR < 50 min^−1^ to avoid too low heart rates. For normal sinus rhythm, pulse lengths *T*_*c*_ are shown to follow the distribution of a (correlated) pink noise [24]. Therefore, *T*_*c*_ are chosen to be realizations of pink noise with the mean 60/HR and the variance *σ*^2^, which varies among the subjects (σ∼N(0.07,2%)).

To consider variations in heart pumping, we vary $E_{\text{fw}}^{\text{max}}$ and *τ*_1_ and *τ*_2_ randomly. For each pulse, we choose

$$\begin{array}{c} E_{\text{fw}}^{\text{max}}∼N\left({\bar{E}}_{\text{fw}}^{\text{max}},P^{\left(s\right)}\%\right),\;\tau_{1}={\bar{\tau }}_{1}c,\;\tau_{2}={\bar{\tau }}_{2}c,\;c∼N\left(1,1\%\right), \end{array}$$ (31)

where $P^{\left(s\right)}∼U\left(0,15\right)$ represents the level of variations in heart muscle contraction between pulses, which is modelled to vary between subjects. The valve model parameters *A*_eff,max_, *A*_eff,min_, *ℓ*_eff,min_, *K*_vo_, and *K*_vc_ are varied with 10% variation.

#### Vascular beds

Microvasculature compliances (*C*) and the reference capillary resistances (*R*_0_ or *R*_0,*m*_) are chosen as $C∼N(\bar{C},5\%)$ and $R_{0}∼\left(1.2{\bar{R}}_{0},20\%\right)$. The mean resistance is increased slightly to provide higher, physiologically more relevant diastolic and systolic pressure levels. For coronary vascular beds (see Fig 3), the resistances *R*_1_ and *R*_2_ and the initial volumes *V*_0,1_ and *V*_0,2_ are perturbed with 10% variation.

#### Generation of the virtual database

We generate two datasets: the first is used to train predictors (training set), and another for the validation of predictions (test set). The generation of the training set is described first.

The model is run repeatedly for the parameter variations described above. The initial state for the solution and the model parameters not specified above are set as in [19]. The 1D-model is discretized using varying number of elements in each segment (*N*_el_ = ⌈0.5*L* ⌉ where *L* is the length of the segment) and the 3rd/2nd order (arteries/veins) Legendre polynomials. The time stepping for temporal discretization is chosen to be Δ*t* = 2 ⋅ 10^−6^ s. The level of discretization is experimentally verified to result sufficiently small discretization error (compared to a very dense discretization). We simulate 11 heart cycles to ensure that the simulation has been converged (e.g. the dependency to the initial condition is negligible) and the last pulse of each run is used in the analysis. The model is run 9986 times. However, we noticed that similar results can also be achieved with significantly less samples (e.g. 1000) and therefore we can assume that the size of database is sufficient.

To ensure that simulations represent physiologically reasonable solutions, the filtering criteria used in [12, 13] are also applied here: a simulation is accepted only if 1) DBP at the brachial arteries are higher than 40 mmHg, 2) SBP at the brachial arteries are lower than 200 mmHg, the pulse pressures (SBP—DBP) at the brachial arteries are between 25-100 mmHg, 4), the reflection coefficient of the aortic-iliac bifurcation satisfies |*R*_*f*_| ≤ 0.3. The reflection coefficient is calculated as

$$\begin{array}{c} R_{f}=\frac{Y_{\text{abd}}-Y_{\text{il,left}}-Y_{\text{il,right}}}{Y_{\text{abd}}+Y_{\text{il,left}}+Y_{\text{il,right}}}, \end{array}$$ (32)

where the characteristic admittances *Y* = *A*_*d*_/(*ρc*_*d*_) (the subscript *d* refers to diastole) are for the distal abdominal aorta (*Y*_abd_) and the proximal common iliac arteries (*Y*_il,left_, *Y*_il,right_).

Out of the 9986 cases, 5222 samples are accepted after applying the above filtering criteria. Out of the rejected samples, 4543 have too small or large reflection coefficient, 70 have to too small diastolic BP, 9 have too large systolic pressure, and pulse pressure is too large for 1115 samples. The large portion of rejected samples due to insufficient reflection constants can perhaps be avoided if more precise information about spatial variations of arterial diameters and stiffness would be available.

The test set is generated similarly, but with a denser discretization (Δ*t* = 0.5 ⋅ 10^−6^ s, 4th/3rd order Legendre polynomials for arteries/veins). This dataset comprises of 943 virtual subjects (1792 before filtering). The training and test set have their own unique virtual patients without overlap.

#### Simulated PPG signal and calculation of PTT/PATs

In this study, we consider predictions based on pulse transit and arrival timings derived from simulated PPG signal. The measurement locations (*x*_obs_) considered in this work are listed in Table 1. PPG signal can be understood as a differential measurement of blood volume under the sensor. If we assume that longitudinal variations in the blood veins are negligible, the blood volume can be assumed to be proportional to *A* (*x*_obs_, *t*). Therefore PPG signal is simulated by removing the scale information:

$$\begin{array}{c} PPG\left(t\right)=\frac{A\left(x_{\text{obs}},t\right)-A_{\text{min}}}{A_{\text{max}}-A_{\text{min}}}. \end{array}$$ (33)

where *A*_min_ and *A*_max_ are the minimum and maximum of *A* (*x*_obs_, *t*) over a period of time. We, however, note that the scale does matter when considering PTT/PAT timings.

**Table 1:** The sensor locations considered in this work. *x*_obs_ is the location of the sensor within the segment and *L* is the length of the segment.

Arteria	Abbreviation	*x*_obs_
Left common carotid artery	LCA	0.65*L*
Right common carotid artery	RCA	0.6*L*
Left/right radialis artery	LRad/RRad	0.9*L*
Right femoral artery	Fem	0.5*L*

**Fig 4:**
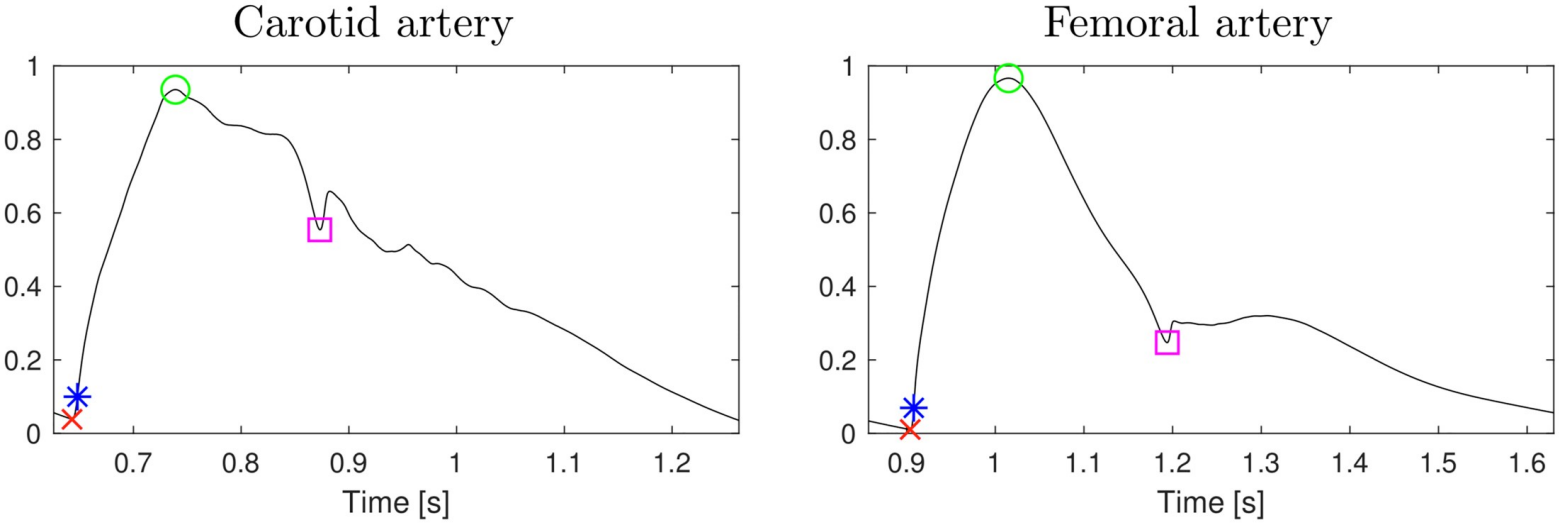
Two example pulses with the considered timings marked: the minimum/foot (PTT_ff_; red cross), the maximum (PTT_p_; green circle), the maximum of the first derivative (PTT_*D*_; blue star), and dicrotic notch (DAT; magenta square).

Arrival of the pulse can be detected as a valley at the beginning of systolic period when pressure *p* (*x*_obs_, *t*) starts increasing (foot-to-toot PTT; PTT_ff_). Other timings can also be considered: the peak (maximum; PTT_p_), the steepest raise (the maximum of the derivate; PTT_*D*_), and the location of the dicrotic notch (DAT); see Fig 4. DAT can be detected as the peak in the second derivate during the diastolic period.

The pulse transit times are relative to aortic valve opening which can be easily detected from simulations: we detect a valley in the simulated pressure *p* (*x*, *t*) at aortic root (the inlet of the 1D segment connecting to aortic valve). For pulse arrival times, simulated R-wave locations can chosen to be the initiation of the pulse (foot) in the prescribed *E*_fw_ for LV.

We note that our simplified PPG signal model does not take into account phenomena such as optical scattering which can induce nonlinear effects to pulse waveform. However, we use PPG signal only to infer timings in the pulse and therefore possible nonlinearities do not have significant effects to results as long as foots, peaks and notches can be estimated accurately. Furthermore, we note that other measurement modalities measuring volume/area of the artery (e.g. ultrasound) can also be considered.

#### Extraction of aPWV, DBP, SBP and SV

Thea aim is to predict aPWV, DBP, SBP or SV using the combination of PTT/PAT times and/or HR (input). These can be extracted from simulated pulses as follows.

- aPWV: the wave speed *c* (*t*, *x*) given by (16) averaged over a pulse (integrated numerically). The location *x* is chosen to be the center point of the segment of aorta between the branching points of brachiocephalic artery and LCA).
- DBP, SBP: the minimum and maximum value of *p* (*x*, *t*) at the aortic root (the inlet of the 1D segment connecting to aortic valve)
- SV: the integral of flow *q* = *AU* at the aortic root over the pulse (calculated numerically).

There are also other options to specify aortic PWV. For example, we can use the foot-to-foot aortic PWV by detecting arrivals of pulses to aortic root and the aortic-iliac bifurcation, but this leads only to very minor differences in the results (the Pearson correlation for between these aPWVs is *r* > 0.99). Relationships between these different options are studied in [12].

**Fig 5:**
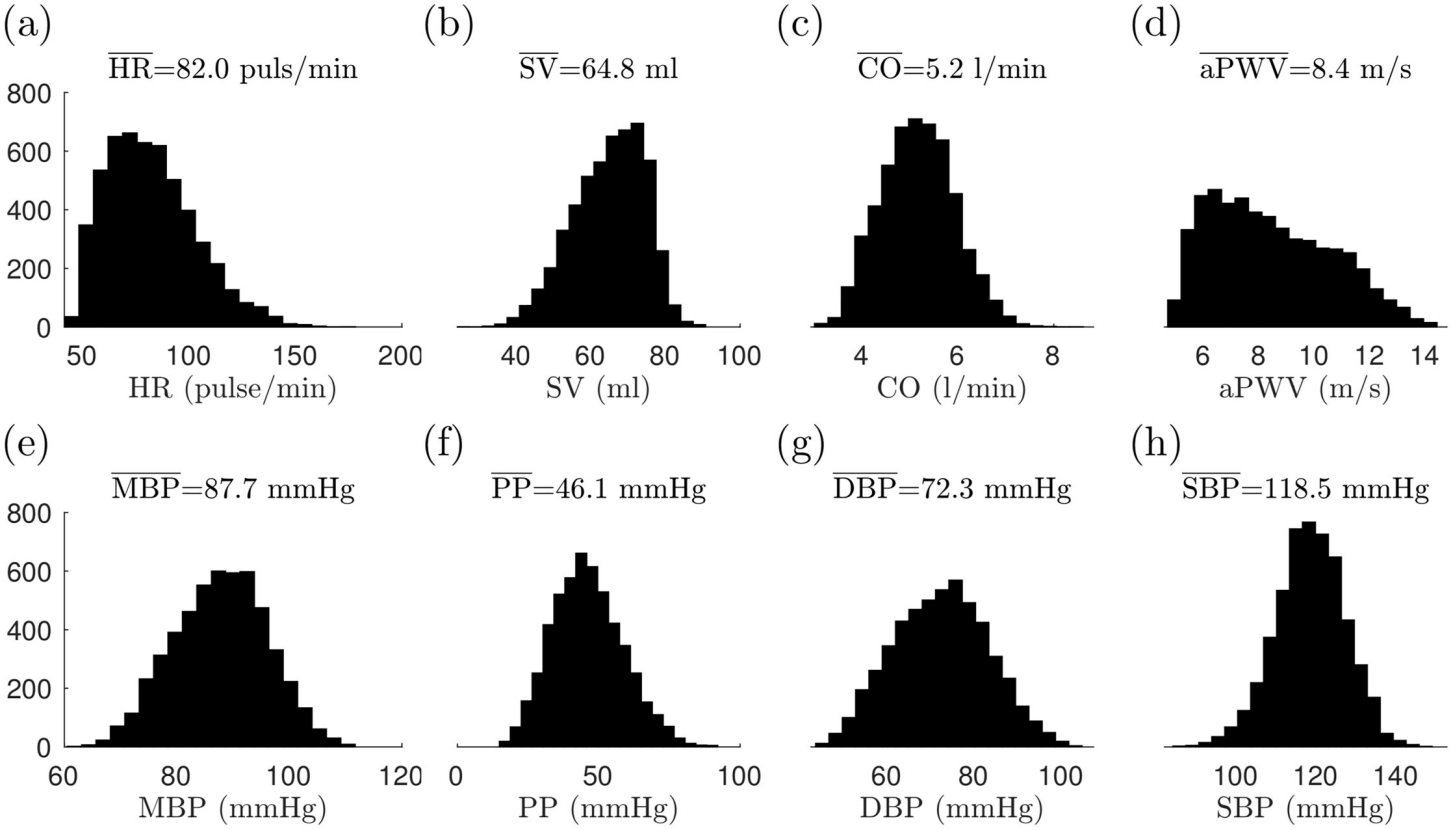
Distributions of selected metrics for the virtual database (training set; after filtering): (a) heart rate (HR), (b) stroke volume (SV), (c) cardiac output (CO), (d) aortic PWV, (e) mean blood pressure (MBP), (f) pulse pressure (PP), (g) diastolic pressure (DPB), and (h) systolic blood pressure (SPB). The means of the metrics are shown in the title.

The distributions of selected metrics of the generated virtual database are shown in Fig 5. As a general finding, we note that there are strong correlations between DAT and pulse length (1/HR) signals (Pearson correlation *r* = 0.96 − 0.98). Due to this strong correlation, using HR and DAT as input provides very similar predictions (which can also be seen in the results below).

### Gaussian process model for predictions

We apply Gaussian process regression for the computation of predictors. GPs are widely used, for example, in machine learning, hydrogeology and analysis of computer experiments (e.g. see [25–27]). GPs also provide flexible predictors that can handle non-linear relationship between input data and the response variable as well as uncertainties in the data. However, we note that any other class of regressions capable of nonlinear relationships can also be used for the analysis. For example, similar results can be achieved with multivariate adaptive regression splines [28].

A GP is a stochastic process *f* (*z*) ($z\in R^{d}$) such that *f* (*z*_1_), …, *f* (*z*_*n*_) is a multivariate Gaussian random variable for all combinations of *z*_1_, …, *z*_*n*_. It can be described by the specifying mean function *μ* (*z*) = **E**(*f* (*z*)) and the covariance function *k* (*z*, *z*′) = cov(*f* (*z*), *f* (*z*′)). For more details, see e.g. [25].

Consider a case in which the inputs *z* are a vector of PTT or PATs and possibly HR and *y* is the response variable (aPWV, DBP, SBP or SV). We model the response variables as

$$\begin{array}{c} y\left(z\right)=h{\left(z\right)}^{T}\beta +f\left(z\right)+\epsilon , \end{array}$$ (34)

where *h* (*z*) is a vector of (deterministic) basis functions, *β* is a vector of basis function coefficients, *f* (*z*) is a GP with zero mean and covariance function *k* (*z*, *z*′), and *ϵ* is an Gaussian white noise. The first term represents mean behavior of the GP model. The GP term models non-linear relationship between input data and the response variable as well as correlated uncertainties in the data.

Training data comprises of input-output pairs {(*z*_*i*_, *y*_*i*_); *i* = 1, …, *N* }. We assume that *y*_*i*_’s are output of the above model i.e. *y*_*i*_ = *y* (*z*_1_). Furthermore, let $Z^{\prime }=\left(z_{1}^{\prime },\ldots ,z_{p}^{\prime }\right)$ be inputs for which we want to calculate predictions. Then *Y* = (*y*_1_, …, *y*_*N*_) and $Y^{\prime }=\left(y\left(z_{1}^{\prime }\right),\ldots ,y\left(z_{p}^{\prime }\right)\right)$ are both Gaussian and the conditional distribution of *Y*′ given *Y* is (see e.g. [25], Appendix A.2]),

$$\begin{array}{c} p\left(Y^{\prime }|Y\right)=N\left(\mu_{Y^{\prime }}+\Sigma_{Y^{\prime }Y}\Sigma_{Y}^{-1}\left(Y-\mu_{Y}\right),\Sigma_{Y^{\prime }}+\Sigma_{Y^{\prime }Y}\Sigma_{Y}^{-1}\Sigma_{YY^{\prime }}\right) \end{array}$$ (35)

where *μ*_*Y*_ and *Σ*_*Y*_ denotes the mean and covariance of *Y* and *Σ*_*YY*′_ is the cross-covariance of *Y* and *Y*′. The means and covariances can be calculated by pluggin in the model (34), which gives

$$\mu_{Y^{\prime }|Y}\;=\;h{\left(Z^{\prime }\right)}^{T}\beta +k\left(Z^{\prime },Z\right){\left(k\left(Z,Z\right)+\sigma_{\epsilon }^{2}I\right)}^{-1}\left(Y-h{\left(Z\right)}^{T}\beta \right)$$ (36)

$$\Sigma_{Y^{\prime }|Y}\;=\;k\left(Z^{\prime },Z^{\prime }\right)-k\left(Z^{\prime },Z\right){\left(k\left(Z,Z\right)+\sigma_{\epsilon }^{2}I\right)}^{-1}k\left(Z,Z^{\prime }\right)$$ (37)

where *h* (*Z*′) and *k* (*Z*′, *Z*) are shorthand notations for the vector and matrix with the components $h(z_{i}^{\prime })$ and $k(z_{i}^{\prime },z_{j})$, respectively. The above conditional mean gives us an prediction of *Y*′ with a confidence estimate given by the conditional covariance.

In this study, the covariance function are chosen to be Matern kernel function with *ν* = 3/2 with a separate length scales for each input parameter. This kernel function can be written as

$$\begin{array}{c} k\left(z,z^{\prime }\right)=\sigma^{2}\left(1+\frac{\sqrt{3}}{r}\right)\text{exp}\left(-\sqrt{3}r\right),\;r={\left(\overset{d}{\underset{m}{\sum }}\frac{{\left(z_{i}-z_{j}\right)}^{2}}{\overset{2}{\underset{m}{\ell }}}\right)}^{1/2} \end{array}$$ (38)

where *σ*^2^ is the variance and *ℓ*_*m*_ are the length scales for each input. We note that the choice of the kernel function does not have a large effect to the results as our sample size is large. For example, our experiments show that use of the squared exponential covariance function gives very similar results with differences of the same scale as the prediction uncertainty.

The predictors are computed using fitrgp function in MATLAB Machine Learning Toolbox which provides numerically efficient implementation for the GP regression. The basis functions *h*(*z*) are chosen to be linear. The fitrgp function also estimates hyperparameters $\theta \;(\beta ,\;\sigma_{\epsilon }^{2},\;\sigma^{2},\;\ell_{1},\;\ldots ,\;\ell_{d})$ by minimizing the negative loglikelihood,

$$\begin{array}{c} L\left(\theta \right)=-\log p\left(y|Z,\theta \right)=\frac{1}{2}y^{T}\Sigma_{\theta }^{-1}y+\frac{1}{2}\log\text{det}\Sigma_{\theta }+\frac{n}{2}\log2\pi \end{array}$$ (39)

where $\Sigma_{\theta }=k\left(Z,Z;\theta \right)+\sigma_{\epsilon }^{2}I$. The optimization is carried out using a subset of observations to avoid high computational load. The parameters of fitrgp related to this hyperparameter optimization are chosen to be the default values.

## Results

In this section, we apply GP regression to predict aPWV, DBP, SBP and SV using combinations of different type of PTT/PAT timings and HR as input. We train a GP predictor separately for each considered combination as described above. For validation, we apply the trained predictor to the test set and calculate Pearson correlation between the predictions and ground truth values. Tables A-H in S1 Appendix also report 95% confidence intervals (CI) for the Pearson correlations (BCa bootstrapping intervals [29]). Each table also highlights selected predictions with largest Pearson correlations. However, we note that the order of Pearson correlations should be considered as indicate rather than a definite order of performance due to the uncertainty especially when differences are small.

### Predictions of aPWV

**Fig 6:**
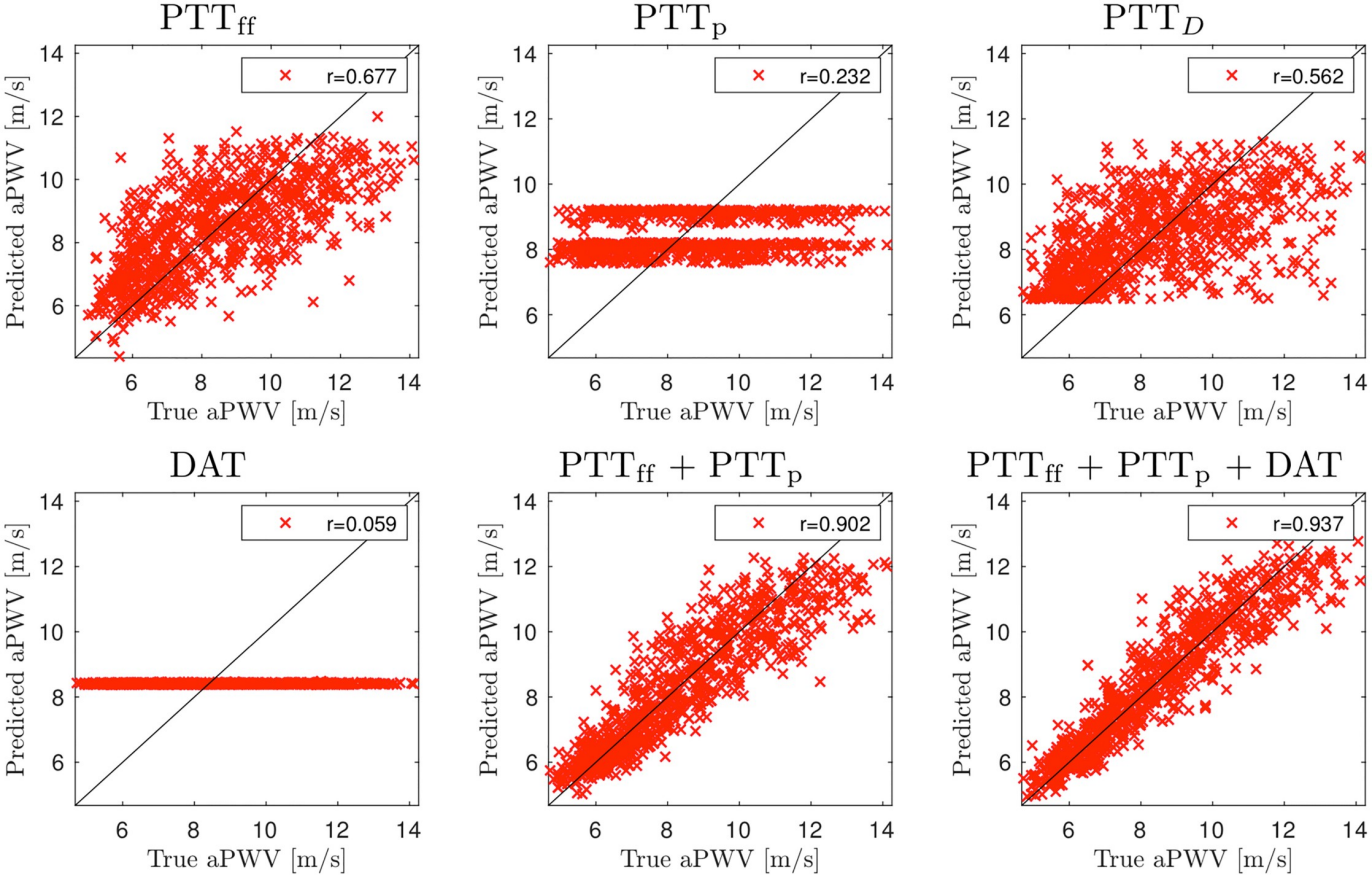
Accuracy of the aortic PWV predictions using pulse transit time (PTT) measurements from left carotid artery (LCA). Signals: heart rate (HR) and pulse transit times to the foot of signal (PTT_ff_), peak of signal (PTT_p_), the point of steepest raise (PTT_*D*_), and the dicrotic notch (DAT).

Fig 6 shows predictions of aPWV for a selected set of combinations when the measurement location is LCA. Table A in S1 Appendix summarizes the results for the complete set of combinations.

The results show that using PTT_ff_ or PTT_*D*_ as a single input gives moderate accuracy and predictions using either HR, PTT_p_, or DAT are insufficient. Performance can be improved by combining multiple different timings. For example, the accuracy is significantly improved if both PTT_ff_ and PTT_p_ are used for predictions (*r* = 0.90). Furthermore, including also DAT provides the accuracy of *r* = 0.94, and adding other timings does not significantly improve accuracy any further.

Measurements from RCA provide less accurate predictions (Table B in S1 Appendix): for example, the combination of PTT_ff_, PTT_p_, PTT_*D*_ and DAT provides one of highest accuracies for RCA (*r* = 0.79), but is still only moderate. Such results can be expected as pulse waves travel shorter distance in aorta and also travel through brachiocephalic artery (see Fig 1) inducing additional variations to the (average) wave speeds.

Performance of wrist measurements (LRad / RRad) are even worse (see Table C in S1 Appendix for LRad; results for RRad are similar). For example, the highest accuracy (*r* = 0.73) can be achieved with the combination of PTT_ff_, PTT_p_, PTT_*D*_ and DAT. This is also expected as relative large part of the arterial tree to these measurement locations are comprised of brachial and radialis arteries with their own variations to PWV. On the other hand, measurements from lower limb could provide better performance: for right femoral artery, we can achieve *r* = 0.75 using PTT_ff_ and *r* = 0.84 using PTT_ff_, PTT_p_, PTT_*D*_ and DAT (Table D in S1 Appendix). In this case, pulse travels though the whole aorta to reach these measurement locations.

As mentioned above, in practice, the R-peak location in ECG signal is often used as a surrogate to aortic valve opening due to simpler measurement. However, using PATs gives only mediocre accuracy compared to PTT due to the physiological variations in PEP [7, 8]. Our finding are similar, see for example, Fig A and Table E in S1 Appendix for LCA. The highest accuracy is *r* = 0.79 (e.g. PAT_ff_, PAT_p_, PAT_*D*_ and HR) which is significantly worse compared to using PTTs.

Another approach to avoid measurement of aortic valve opening is to consider differences of pulse arrival times to two distal locations. Such setup also allows us to avoid the influence of PEP variations. Results for measurement between LCA and Fem can be seen in Fig B and Table F in S1 Appendix: difference of PTT_ff_ gives *r* = 0.76 which is slightly better than using normal PTT_ff_ measurement from Fem, but not as good as normal PTT_ff_ measurement from LCA. The highest accuracy (*r* = 0.87) can be obtained, for example, with PTT_ff_, PTT_p_, PTT_*D*_ and HR. The predictions of PWV that use the difference between LCA and RCA or the difference between LRad and RRad are less accurate (*r* ≈ 0.75 − 0.78 at best); see Tables G and H in S1 Appendix.

### Predictions for blood pressure

**Fig 7:**
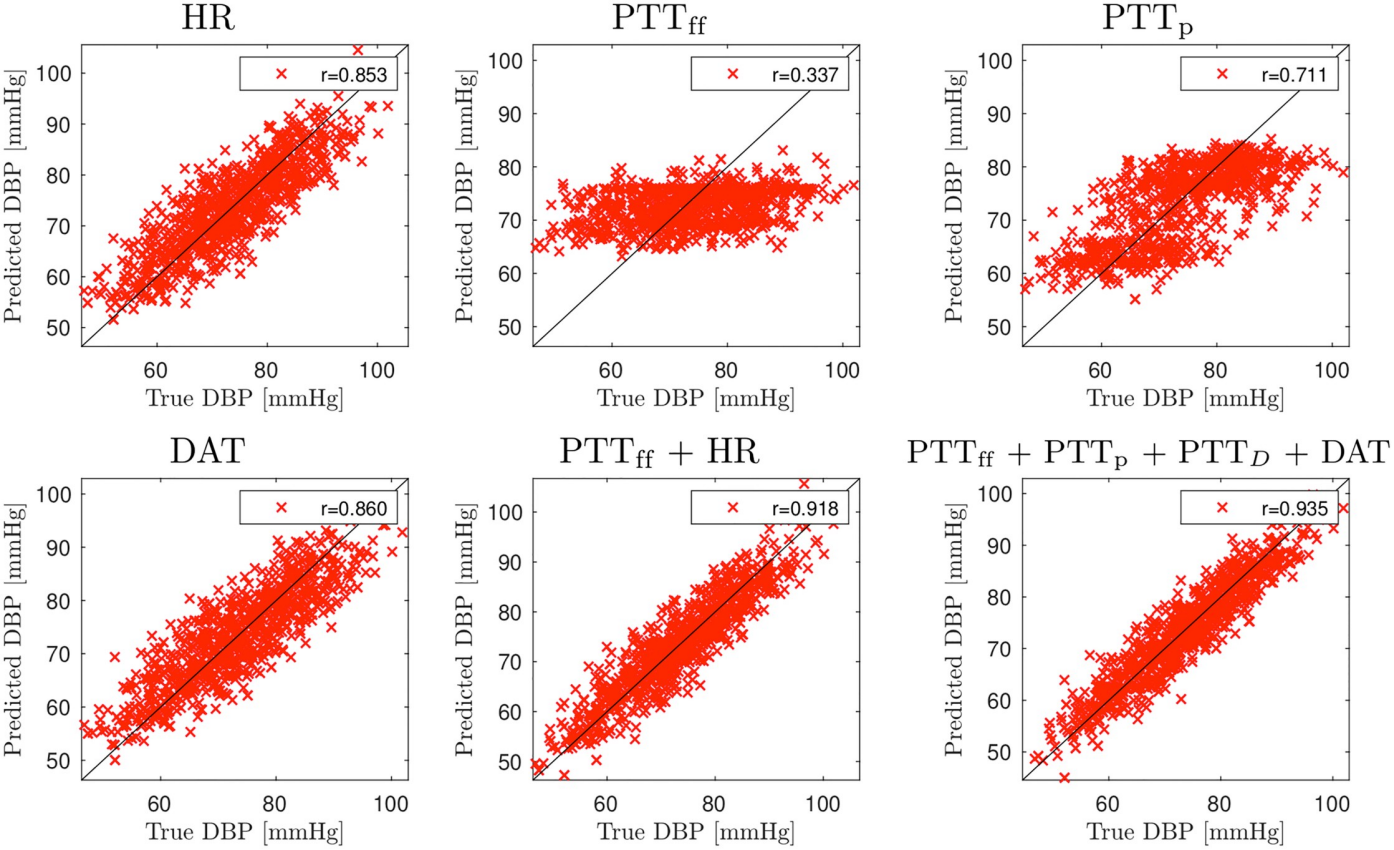
Accuracy of the DBP predictions using pulse transit time (PTT) measurements from left carotid artery (LCA). Signals: heart rate (HR) and pulse transit times to the foot of signal (PTT_ff_), peak of signal (PTT_p_), the point of steepest raise (PTT_*D*_), and the dicrotic notch (DAT).

**Fig 8:**
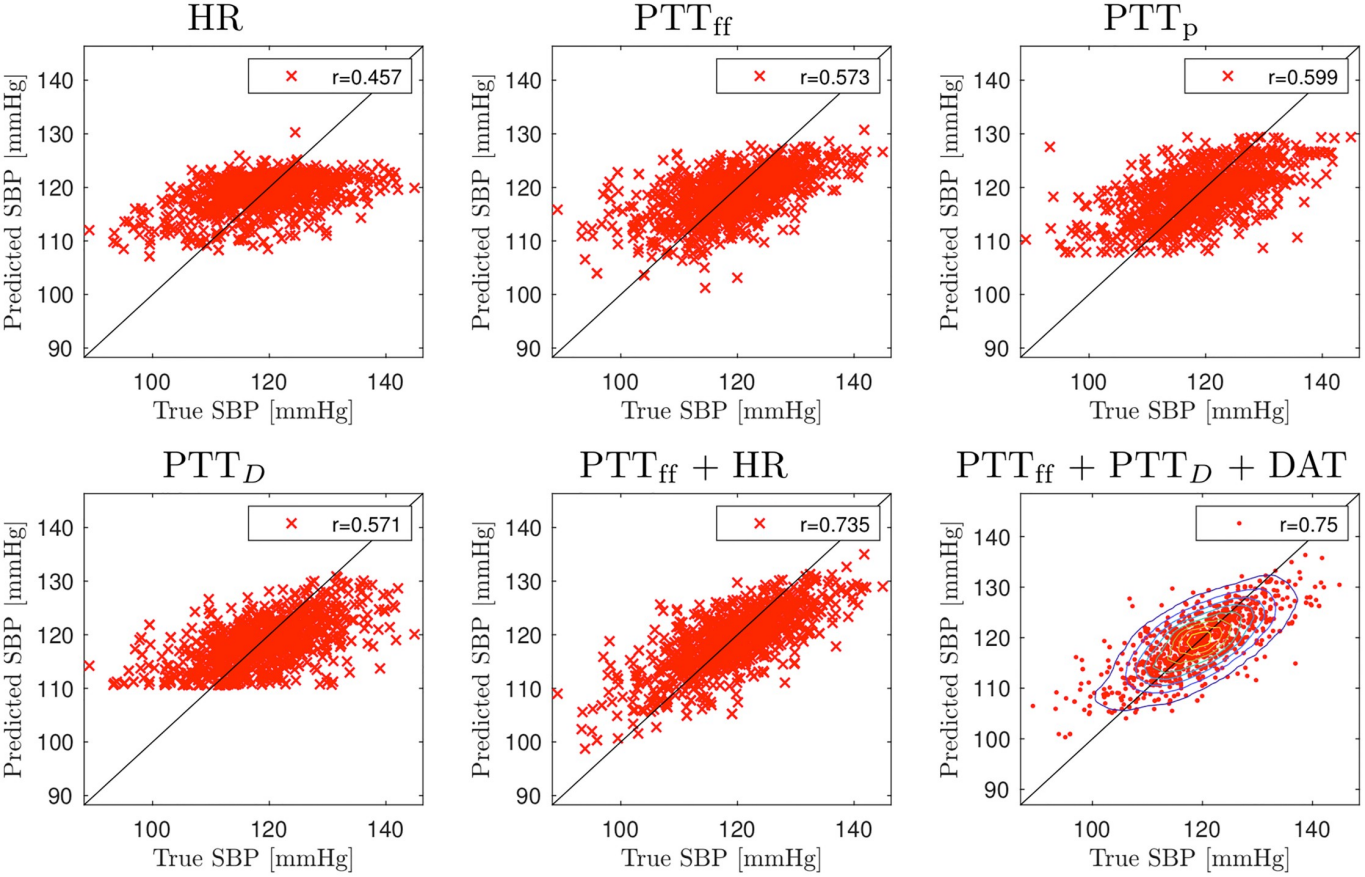
Accuracy of the SBP predictions using pulse transit time (PTT) measurements from left carotid artery (LCA). Signals: heart rate (HR) and pulse transit times to the foot of signal (PTT_ff_), peak of signal (PTT_p_), the point of steepest raise (PTT_*D*_), and the dicrotic notch (DAT).

Figs 7 and 8 show predictions for DBP and SBP for selected PTT time combinations when measurements are taken from LCA; see also Table A in S1 Appendix for all combinations. For DBP, predictions using PTT_ff_ as a single input achieves very low accuracy (*r* = 0.33). Significantly more accurate predictions can be achieved using HR (*r* = 0.85) or DAT (*r* = 0.86). For SBP, the performance of PTT based predictions is better but still quite low (*r* = 0.58 for PTT_ff_ and *r* = 0.60 for PTT_p_). Predictions can be improved by adding additional input timings. For DBP, combining PTT_ff_ with HR or DAT gives *r* = 0.92 and the highest accuracy *r* = 0.94 is obtained with PTT_ff_, PTT_p_, PTT_*D*_ and DAT. Additional input timings also improves performance of SDB predictions: PTT_ff_ and HR/DAT results in *r* = 0.735 and the highest accuracy is *r* = 0.75 (PTT_ff_, PTT_p_, PTT_*D*_ and DAT). Findings the other measurements locations are similar; see Tables B, C and D in S1 Appendix.

We also consider predictions from pulse arrival times (i.e. using R-peak as a reference timing). Compared to PTT times, the results are of mixed accuracy; see Table E for PAT measurements from LCA. For DBP, using PAT_ff_ as single input yields insufficient predictions (*r* = 0.19), but PAT_p_ gives moderate accuracy (*r* = 0.67). Combinations of different PAT timings can even achieve higher accuracy than using PTTs: for example, *r* = 0.95 with PAT_ff_ and DAT and *r* = 0.96 for PAT_ff_, PAT_p_, PAT_*D*_ and DAT. For SBP, PAT_ff_ provides slightly better accuracy compared to PTT_ff_(*r* = 0.62), but otherwise results are similar.

As with aPWV, we consider differences of pulse transit/arrival times measured with two sensor. Measuring between LCA and Fem gives very similar performance to PTT measurements from LCA (Table F in S1 Appendix). However, other considered setups provide less accurate results: see Table G in S1 Appendix for differences between LCA and RCA measurements and Table H for differences between measurements from radialis arteries.

### Prediction of SV

Results show that HR has largest contribution to the predictions of SV, meanwhile performance with pulse transit or arrival timings (without HR information) can only provide moderate accuracy at best. For example, Fig G and Table A in S1 Appendix show the predictions using measurements from LCA. Predictions with HR as a single input reaches *r* = 0.81, but predictions using PTT_ff_ or PTT_*D*_ are insufficient estimates (*r* < 0.25) and predictions with PTT_p_ are of moderate accuracy (*r* = 0.60). SV can be predicted with good accuracy with DAT, but this is due to the strong correlation between HR and DAT as mentioned above. Furthermore, significant improvements will not be achieved by combining several inputs. For example, highest accuracy is *r* = 0.83 which can be obtained, for example, with PTT_ff_, PTT_*D*_ and HR. Results are similar for all other measurement setups; see Tables B-H in S1 Appendix.

## Discussion

This paper assessed theoretical limitations for the prediction of aortic pulse wave velocity (aPWV), DBP/SBP and SV from pulse transit and arrival time measurements. We applied a virtual database approach proposed by Willemet et al [12, 13] in which a cardiovascular simulator is used to generate a database of virtual subjects. In this work, we applied one-dimensional haemodynamic model by Mynard and Smolich [19] to construct a simulator for entire adult circulation. This simulator was used to generate a large database of synthetic blood circulations with varied physiological model parameters. The generated database was then used as training data for Gaussian process regressors. Finally, these trained regressors were applied to another synthetic database (test set) to assess capability of regressors to predict aPWV, SDB, DBP and SV using different combinations pulse transit/arrival time and HR measurements.

The results indicate that aPWV and DBP can be estimated from PPG signal with a high accuracy (Pearson correlation *r* > 0.9 between true and predicted values for measurement from left carotid artery) when, in addition to foot-to-foot PTT time, information about the peak and dicrotic notch location is also given as input to the predictor. The predictions of SDB were less accurate (*r* = 0.75 at best). For SV, accurate predictions were mostly based on heart rate, with only a very minor improvement in accuracy when PTT timings were also included as inputs.

As this was entirely in silico study, it is not guaranteed that the result can be applicable to the real world as is. However, the aim of the study was to give preliminary results about correlations between the cardiac indices and PTT/PAT timings and the applicability of such predictions. The hope is that the results could to be extended to real clinical applications in future research.

The limitations to be addressed in future are the following. First, the cardiovascular model has its limitations. Although previous studies have shown that similar cardiovascular models can be used to simulate human physiology relatively well [16–18], not all physiological phenomena are fully covered in the Mynard’s model. One example of such phenomenon is respiration. The effect of respiration can be important as the breathing and cardiac cycles are in a close interaction. Several physiological factors, such as the changes in the intrathoracic pressure and the variation in the interbeat intervals modulate the cardiac mechanics and blood outflow from the heart. Even the timing of the shorter cardiac cycles coupled with the longer respiratory cycles has effects on the central circulation. When we considering a healthy heart, the effects of respiration can perhaps be managed by interpreting different virtual subjects to represent inspiratory and expiratory phases of the breathing. Other phenomena that are not covered by the model are, for example, gravity and baroreceptors. Furthermore, lumped parameter models that are used for heart and vascular beds were relatively simple approximations. However, new analytical methods allow us to bridge the models and human bodily functions [30].

The chosen baselines and variations of the model parameters were chosen to represent healthy subject. The choices, however, can be subjective due to the limited amount of (probabilistic) information. Our attempt were to produce variations such that the virtual population covered by the chosen parameter variations includes real physiological variations. We, however, emphasize that the presented approach is not limited to the chosen parameters variations and it can be adjusted if more precise information becomes available.

Due to the limited phenomena covered by the model, the results may not be reliable when considering subjects with medical conditions. For example, the simplified heart model and variations of related model parameter may not present subjects with heart diseases.

In this study, we only considered pulse transit and arrival type of time information as the input to the predictor. Predictions could potentially be improved with other kinds of additional information. For example, aortic PWV predictions could be improved by using information about the distances between aorta and/or measurement points. Information about arterial path lengths could have been easily used in our simulation analysis, but in practice such information would require clinical measurements such as MRI [21, 22]. On the other hand, the arterial path length are often estimated using the body lengths or measuring distances of certain points in the body [21, 22]. Such information was not used in this simulation study as precise statistical knowledge of connection between such body measurement and arterial length was not available. Instead, Gaussian process regressors implicitly marginalize predictions over different arterial lengths that are present in the virtual database.

Ultimately it would be beneficial to develop approaches that do not need reference measurement (aortic valve opening/R-peak). For example, Choudhury et al [31] presented a machine learning algorithm which uses raise times and pulse widths derived from PPG signal to predict DBP and SBP. Furthermore, deep learning approaches could perhaps be used to infer optimal information from PPG waveform. These are subject of our future research.

## Supporting information

S1 Appendix Additional results. Electronic supplementary material reporting additional numerical findings.
(PDF)
