# Supplementary material for: Pulse transit time estimation of aortic pulse wave velocity and blood pressure using machine learning and simulated training data
Source: PLoS Comput Biol. 2019 Aug 15;15(8):e1007259. doi: 10.1371/journal.pcbi.1007259 (PMC6711549; doi:10.1371/journal.pcbi.1007259)
Supplement: S1 Appendix — Electronic supplementary material reporting additional numerical findings. (PDF) [file pcbi.1007259.s001.pdf]

Supplementary Material for: Pulse transit time estimation of aortic pulse wave velocity and blood pressure using machine learning and simulated training data, by J.M.J. Huttunen, L. Kärkkäinen and H. Lindholm. Corresponding author: J.M.J. Huttunen, janne.m.huttunen@nokia-bell-labs.com

Additional result: figures and tables

This supplementary document expands numerical results with additional figures and tables. Section A.1 presents additional predictions for aortic PWV. Additional predictions for blood pressures are shown in Section A.2 and predictions for stroke volume are shown in A.3.

All results are collected to the tables presented Section A.4. The confidence intervals (CI) that are computed using bootstrapping as follows. A bootstrapping dataset is formed by resampling or picking samples from the training using sampling with replacement. The GP regressor is then trained using this bootstrapping dataset. The results are computed using as previously using a bootstrapped test set (resampled with replacement from the original test set). This procedure is repeated 500 times. The CIs are computed as bias-corrected and accelerated (BCa) intervals [1,2]. The related acceleration parameter is commonly estimated using the jackknife resampling which is similar to bootstrapping except resampled datasets are formed by excluding samples one-by-one. However, in our study, the original jackknife would be computationally very expensive as resampling would be repeated 5222 times. Therefore, we used "partial" jackknife resampling in which the procedure is repeated 500 times such that each time an excluded sample is chosen randomly. It is to be noted that in some rare cases CIs do not include the actual estimate. This is a property of bootstrapping intervals as bootstrapping sets include less independent samples (random sampling with replacement takes roughly 3000-3300 independent samples from the training set of 5222 samples) and none of GPs computed using the bootstrapping datasets do not reach the same accuracy as the GP model trained using the original training set.

References

[1] Efron B. Better Bootstrap Confidence Intervals. Journal of the American Statistical Association. 1987;82(397):171–185.

[2] Fox J. Bootstrapping regression models; 2002. An R and S-PLUS Companion to Applied Regression: A Web Appendix to the Book. Sage, Thousand Oaks, CA.

A.1 Additional figures for prediction of aortic PWV velocity

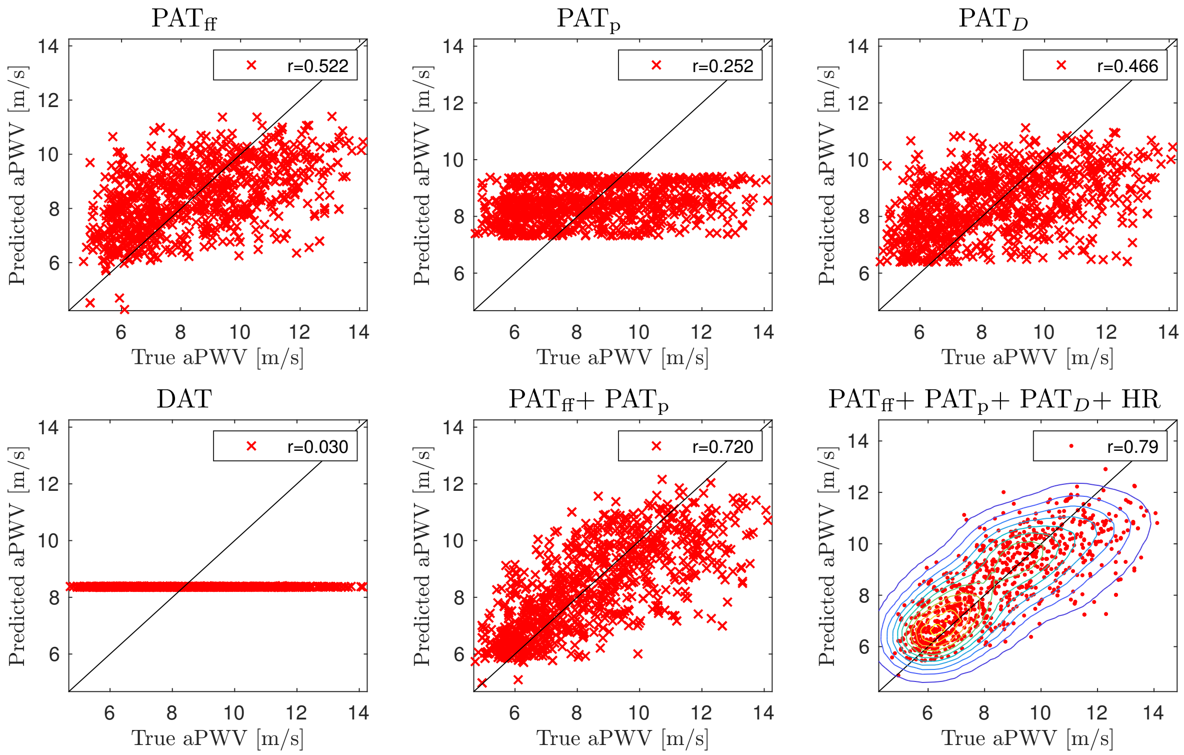

Figure A: Accuracy of aortic PWV predictions using pulse arrival time (PAT) measurements from left carotid artery (LCA). Signals: heart rate (HR) and pulse transit times to the foot of signal (PAT_{ff}), peak of signal (PAT_p), the point of steepest raise (PAT_D), and the dicrotic notch (DAT).

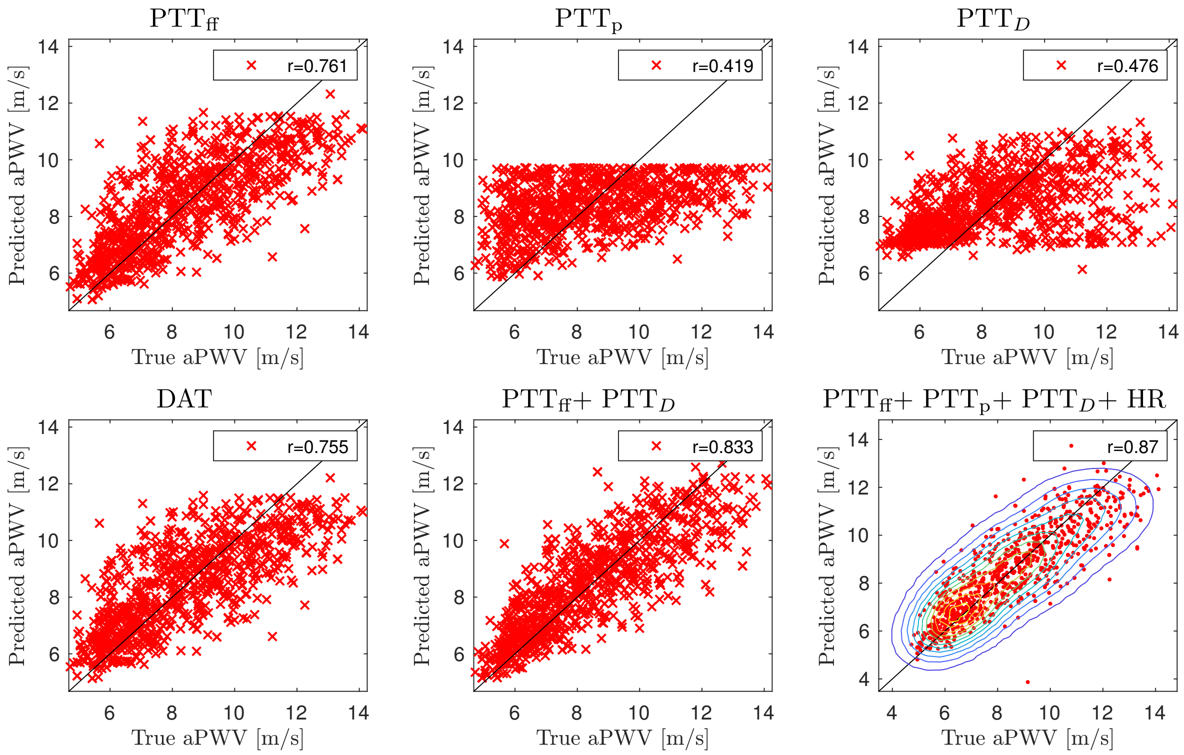

Figure B: Accuracy of aortic PWV predictions using time difference of measurements from LCA and femoral arteries. Signals: heart rate (HR) and pulse arrival times to the foot of signal ($\mathrm{PTT_{ff}}$), peak of signal ($\mathrm{PTT_p}$), the point of steepest raise (PTT_D), and the dicrotic notch (DAT).

A.2 Additional figures for predictions of blood pressure levels

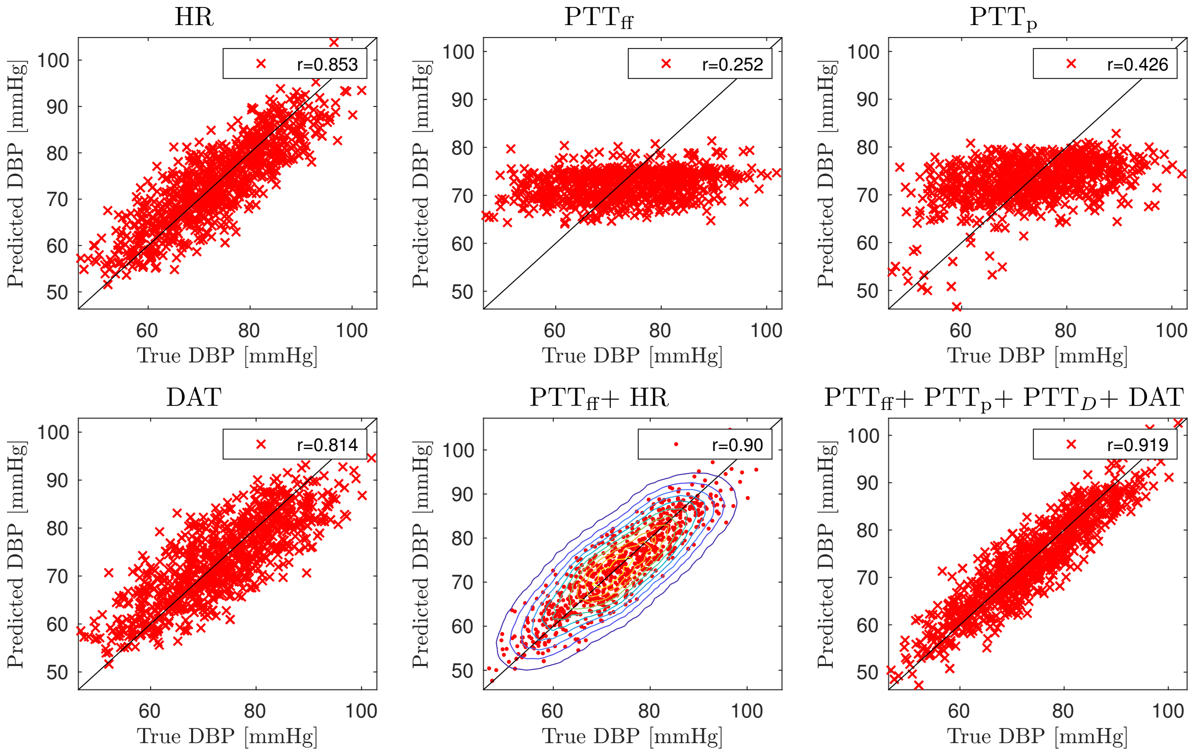

Figure C: Accuracy of diastolic blood pressure (DBP) predictions using pulse transit time (PTT) measurements from left radial artery (LRad). Signals: heart rate (HR) and pulse transit times to the foot of signal ($\mathrm{PTT_{ff}}$), peak of signal ($\mathrm{PTT_p}$), the point of steepest raise (PTT_D), and the dicrotic notch (DAT).

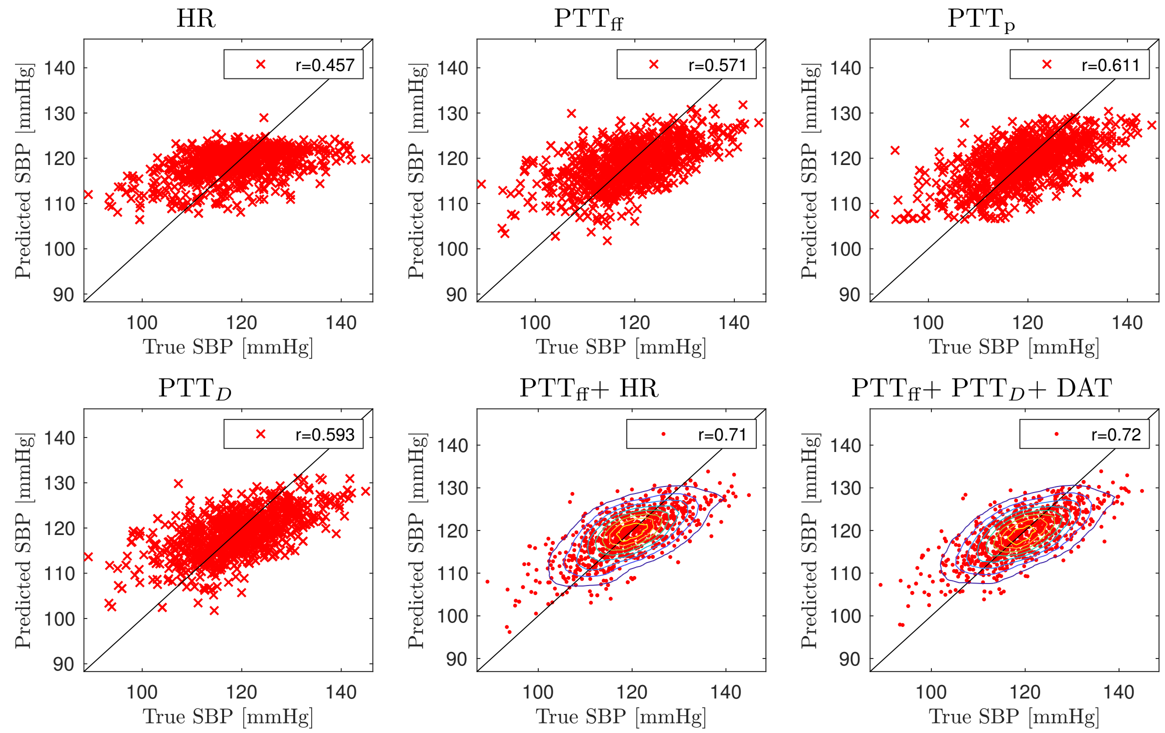

Figure D: Accuracy of systolic blood pressure (SBP) predictions using pulse transit time (PTT) measurements from left carotid artery (LCA). Signals: heart rate (HR) and pulse transit times to the foot of signal (PTT$_{ff}$), peak of signal (PTT$_{p}$), the point of steepest raise (PTT$_{D}$), and the dicrotic notch (DAT).

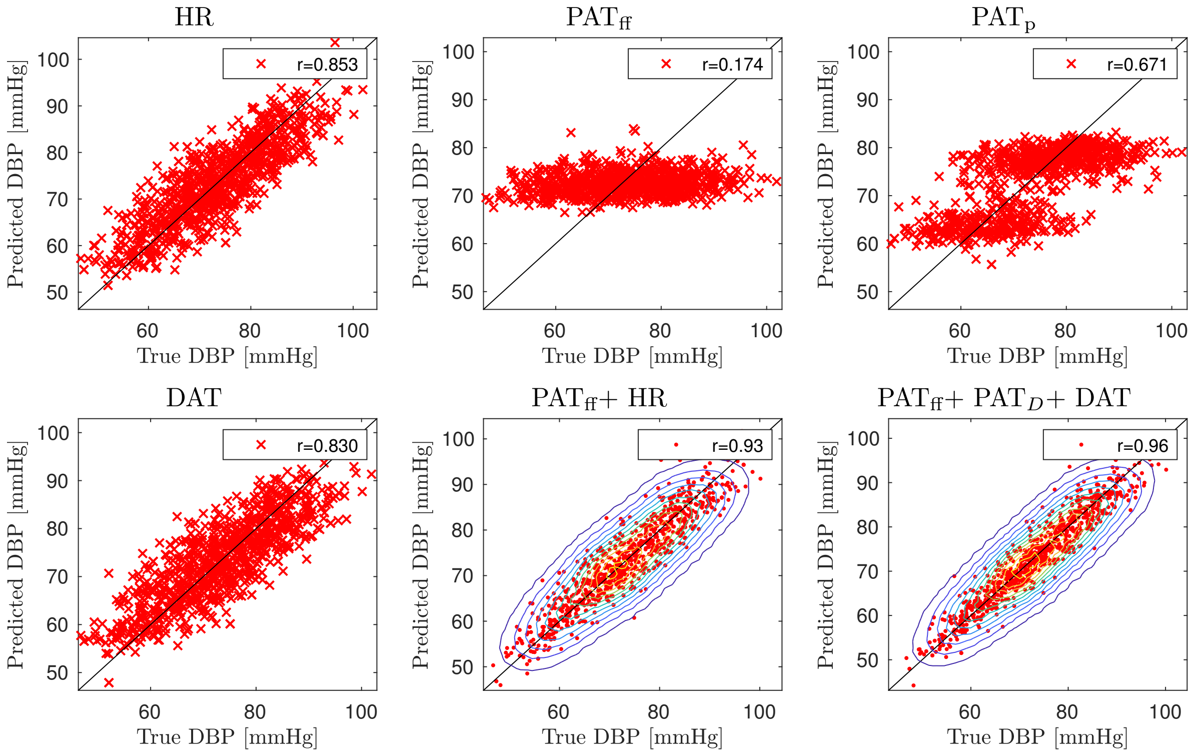

Figure E: Accuracy of diastolic blood pressure (DBP) predictions using pulse arrival time (PAT) measurements from left carotid artery (LCA). Signals: heart rate (HR) and pulse transit arrival to the foot of signal (PAT$_{ff}$), peak of signal (PAT$_{p}$), the point of steepest raise (PAT$_{D}$), and the dicrotic notch (DAT).

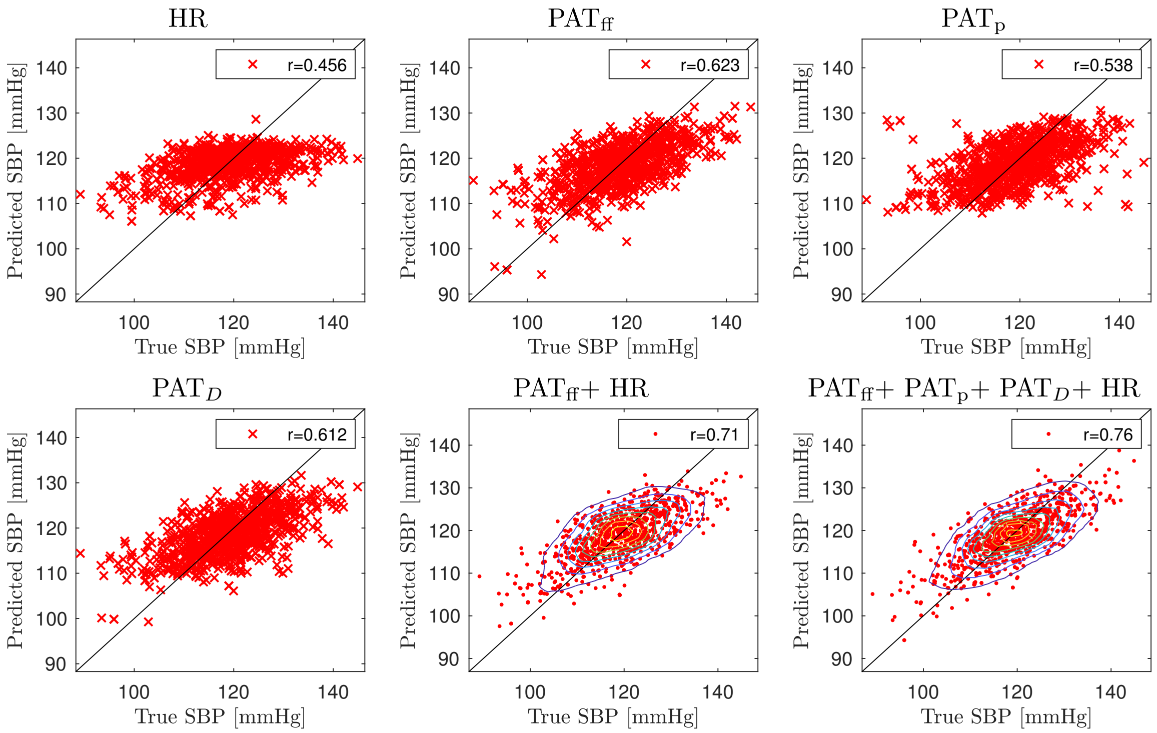

Figure F: Accuracy of systolic blood pressure (DBP) predictions using pulse arrival time (PAT) measurements from left carotid artery (LCA). Signals: heart rate (HR) and pulse transit arrival to the foot of signal (PAT$_{ff}$), peak of signal (PAT$_p$), the point of steepest raise (PAT$_D$), and the dicrotic notch (DAT).

A.3 Additional figures for prediction of stroke volume

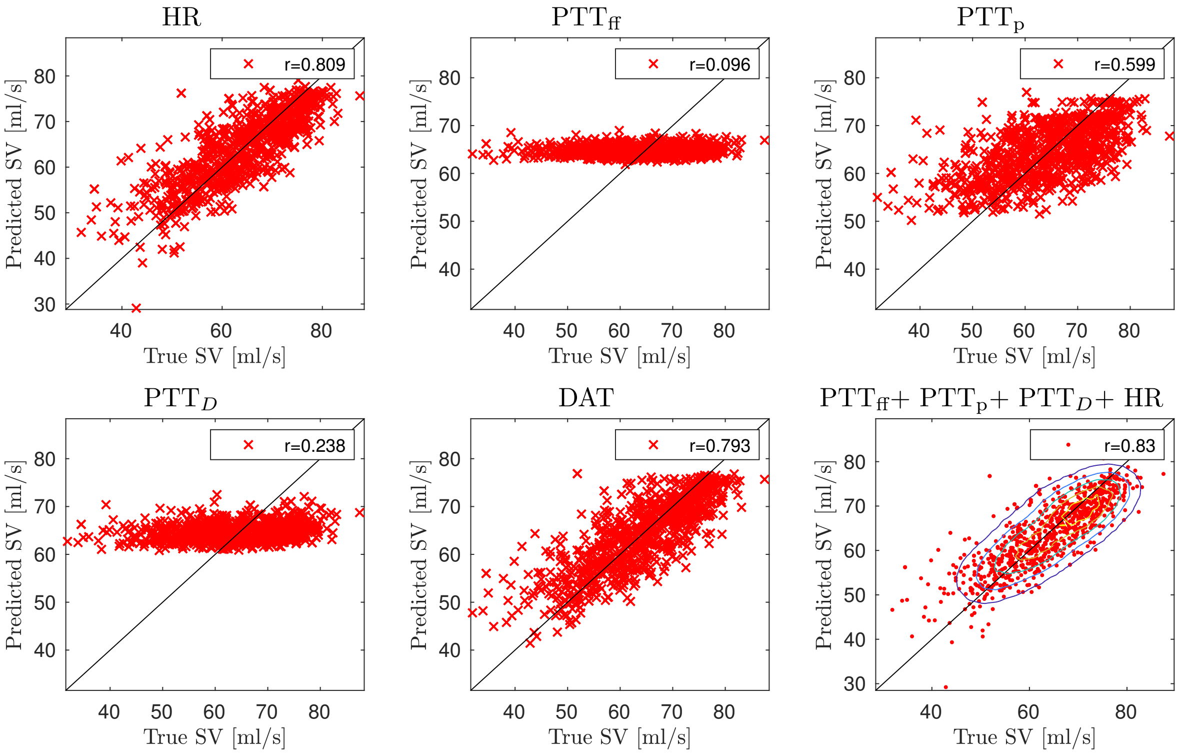

Figure G: Accuracy of the stroke volumen (SV) predictions using pulse transit time (PTT) measurements from left carotid artery (LCA). Signals: heart rate (HR) and pulse transit times to the foot of signal (PTT$_{ff}$), peak of signal (PTT$_p$), the point of steepest raise (PTT$_D$), and the dicrotic notch (DAT).

A.4 Tables

Table A: Accuracy of predictions using PTT measurements (the reference time is the aortic valve opening) from left carotid artery (LCA). The accuracy is expressed as Pearson correlation coefficients with 95% CI (BCa intervals [1]). The predictions with largest Pearson correlations are highlighted: one signal (red), two signals (blue) and five most accurate combination (black). Signals: heart rate (HR) and pulse transit times to the foot of signal (PTT$_{ff}$), peak of signal (PTT$_{p}$), the point of steepest raise (PTT$_{D}$), and the dicrotic notch (DAT).

	aPWV	DBP	SBP	SV
HR	0.06 [-0.08,0.14]	0.85 [0.83,0.87]	0.46 [0.39,0.51]	**0.81** [0.77,0.84]
PTT$_{ff}$	**0.68** [0.64,0.71]	0.33 [0.27,0.40]	0.58 [0.52,0.63]	0.10 [0.02,0.17]
PTT$_{p}$	0.23 [0.15,0.31]	0.71 [0.68,0.74]	**0.60** [0.54,0.65]	0.60 [0.55,0.65]
PTT$_{D}$	0.56 [0.51,0.61]	0.08 [0.00,0.17]	0.57 [0.52,0.62]	0.24 [0.07,0.30]
DAT	0.06 [-0.08,0.13]	**0.86** [0.84,0.88]	0.46 [0.40,0.52]	0.79 [0.76,0.82]
PTT$_{ff}$+HR	0.68 [0.64,0.72]	0.92 [0.90,0.93]	0.74 [0.69,0.77]	0.82 [0.79,0.84]
PTT$_{p}$+HR	0.31 [0.25,0.39]	0.89 [0.87,0.90]	0.67 [0.63,0.72]	**0.82** [0.80,0.86]
PTT$_{D}$+HR	0.59 [0.54,0.64]	0.89 [0.87,0.91]	0.65 [0.61,0.70]	0.81 [0.78,0.84]
DAT+HR	-0.00 [-0.17,0.07]	0.86 [0.84,0.88]	0.45 [0.39,0.51]	0.81 [0.78,0.84]
PTT$_{ff}$+PTT$_{p}$	**0.90** [0.89,0.92]	0.79 [0.76,0.82]	0.69 [0.65,0.73]	0.62 [0.58,0.67]
PTT$_{ff}$+PTT$_{p}$+HR	0.93 [0.92,0.95]	0.92 [0.90,0.93]	0.74 [0.71,0.78]	0.82 [0.80,0.85]
PTT$_{ff}$+PTT$_{D}$	0.72 [0.66,0.76]	0.79 [0.77,0.82]	0.64 [0.60,0.71]	0.54 [0.49,0.59]
PTT$_{ff}$+PTT$_{D}$+HR	0.75 [0.72,0.79]	0.93 [0.91,0.94]	0.74 [0.73,0.76]	**0.83** [0.81,0.86]
PTT$_{ff}$+DAT	0.68 [0.64,0.71]	**0.92** [0.91,0.94]	**0.74** [0.71,0.78]	0.80 [0.77,0.83]
PTT$_{ff}$+DAT+HR	0.68 [0.64,0.72]	0.92 [0.91,0.94]	0.74 [0.71,0.79]	0.81 [0.79,0.84]
PTT$_{ff}$+PTT$_{p}$+PTT$_{D}$	0.92 [0.91,0.93]	0.87 [0.85,0.89]	0.71 [0.69,0.70]	0.70 [0.67,0.74]
PTT$_{ff}$+PTT$_{p}$+PTT$_{D}$+HR	**0.93** [0.93,0.95]	**0.93** [0.92,0.94]	0.74 [0.73,0.76]	**0.82** [0.80,0.86]
PTT$_{ff}$+PTT$_{p}$+DAT	**0.94** [0.93,0.95]	0.92 [0.91,0.94]	**0.75** [0.73,0.79]	0.80 [0.77,0.84]
PTT$_{ff}$+PTT$_{p}$+DAT+HR	**0.94** [0.93,0.95]	0.92 [0.91,0.94]	0.75 [0.72,0.78]	0.82 [0.79,0.85]
PTT$_{ff}$+PTT$_{D}$+DAT	0.75 [0.72,0.79]	**0.93** [0.91,0.95]	**0.75** [0.73,0.80]	0.81 [0.80,0.84]
PTT$_{ff}$+PTT$_{D}$+DAT+HR	0.75 [0.72,0.78]	**0.93** [0.92,0.95]	**0.75** [0.72,0.80]	**0.83** [0.81,0.86]
PTT$_{ff}$+PTT$_{p}$+PTT$_{D}$+DAT	**0.94** [0.93,0.95]	**0.94** [0.92,0.95]	**0.75** [0.73,0.79]	0.81 [0.80,0.82]
PTT$_{ff}$+PTT$_{p}$+PTT$_{D}$+DAT+HR	**0.94** [0.93,0.95]	**0.94** [0.93,0.95]	**0.75** [0.72,0.80]	**0.83** [0.82,0.86]

Table B: Accuracy of predictions using PTT measurements (the reference time is the aortic valve opening) from right carotid artery (RCA). Otherwise same caption as in Table A.

	aPWV	DBP	SBP	SV
HR	0.06 [-0.07,0.14]	0.85 [0.83,0.87]	0.46 [0.39,0.51]	**0.81** [0.77,0.84]
PTT$_{ff}$	**0.60** [0.54,0.64]	0.30 [0.24,0.37]	**0.57** [0.52,0.62]	0.11 [0.03,0.20]
PTT$_{p}$	0.24 [0.18,0.32]	0.78 [0.75,0.81]	0.45 [0.39,0.51]	0.63 [0.58,0.68]
PTT$_{D}$	0.48 [0.42,0.54]	-0.01 [-0.13,0.06]	0.56 [0.47,0.62]	0.27 [0.12,0.34]
DAT	0.07 [-0.03,0.13]	**0.86** [0.84,0.88]	0.45 [0.37,0.51]	0.79 [0.76,0.82]
PTT$_{ff}$+HR	0.60 [0.56,0.65]	0.91 [0.90,0.93]	0.72 [0.68,0.76]	0.81 [0.79,0.84]
PTT$_{p}$+HR	0.31 [0.23,0.40]	0.88 [0.86,0.90]	0.66 [0.62,0.70]	**0.82** [0.80,0.85]
PTT$_{D}$+HR	0.52 [0.47,0.58]	0.89 [0.87,0.91]	0.64 [0.59,0.68]	0.81 [0.78,0.84]
DAT+HR	0.06 [-0.01,0.17]	0.86 [0.84,0.88]	0.45 [0.41,0.52]	0.81 [0.78,0.84]
PTT$_{ff}$+PTT$_{p}$	**0.71** [0.68,0.76]	0.84 [0.82,0.87]	0.69 [0.66,0.73]	0.68 [0.65,0.72]
PTT$_{ff}$+PTT$_{p}$+HR	**0.74** [0.72,0.78]	0.91 [0.90,0.93]	**0.74** [0.73,0.77]	**0.82** [0.80,0.85]
PTT$_{ff}$+PTT$_{D}$	0.66 [0.60,0.71]	0.76 [0.72,0.79]	0.63 [0.57,0.69]	0.49 [0.44,0.56]
PTT$_{ff}$+PTT$_{D}$+HR	0.72 [0.67,0.75]	0.92 [0.91,0.94]	0.73 [0.72,0.76]	**0.83** [0.81,0.85]
PTT$_{ff}$+DAT	0.60 [0.56,0.65]	**0.92** [0.90,0.93]	**0.72** [0.68,0.76]	0.80 [0.77,0.83]
PTT$_{ff}$+DAT+HR	0.59 [0.55,0.64]	0.92 [0.91,0.93]	0.72 [0.69,0.76]	0.81 [0.78,0.84]
PTT$_{ff}$+PTT$_{p}$+PTT$_{D}$	**0.77** [0.76,0.77]	0.87 [0.85,0.89]	0.70 [0.70,0.71]	0.71 [0.69,0.76]
PTT$_{ff}$+PTT$_{p}$+PTT$_{D}$+HR	**0.78** [0.75,0.76]	**0.92** [0.92,0.94]	0.73 [0.73,0.75]	**0.83** [0.81,0.85]
PTT$_{ff}$+PTT$_{p}$+DAT	0.74 [0.71,0.77]	0.92 [0.90,0.93]	0.73 [0.71,0.77]	0.80 [0.77,0.84]
PTT$_{ff}$+PTT$_{p}$+DAT+HR	0.73 [0.73,0.76]	0.92 [0.91,0.93]	0.74 [0.71,0.77]	0.82 [0.79,0.85]
PTT$_{ff}$+PTT$_{D}$+DAT	0.71 [0.68,0.74]	**0.93** [0.91,0.94]	**0.74** [0.72,0.77]	0.80 [0.77,0.83]
PTT$_{ff}$+PTT$_{D}$+DAT+HR	0.71 [0.68,0.74]	**0.93** [0.92,0.94]	**0.74** [0.71,0.77]	**0.83** [0.81,0.85]
PTT$_{ff}$+PTT$_{p}$+PTT$_{D}$+DAT	**0.79** [0.75,0.76]	**0.93** [0.92,0.94]	**0.74** [0.72,0.78]	0.80 [0.80,0.84]
PTT$_{ff}$+PTT$_{p}$+PTT$_{D}$+DAT+HR	**0.79** [0.76,0.77]	**0.93** [0.92,0.94]	**0.74** [0.71,0.77]	**0.83** [0.81,0.85]

Table C: Accuracy of predictions using PTT measurements (the reference time is the aortic valve opening) from left radialis artery (LRad). Otherwise same caption as in Table A.

	aPWV	DBP	SBP	SV
HR	0.06 [-0.07,0.15]	**0.85** [0.83,0.87]	0.46 [0.40,0.51]	**0.81** [0.76,0.84]
PTT_{ff}	**0.33** [0.25,0.39]	0.25 [0.18,0.32]	0.57 [0.52,0.62]	0.16 [0.06,0.23]
PTT_p	0.06 [-0.02,0.13]	0.43 [0.37,0.48]	**0.61** [0.55,0.65]	0.58 [0.53,0.63]
PTT_D	0.32 [0.23,0.38]	0.19 [0.11,0.27]	0.59 [0.52,0.64]	0.20 [0.11,0.27]
DAT	0.04 [-0.04,0.15]	0.81 [0.79,0.84]	0.52 [0.46,0.57]	0.80 [0.77,0.83]
PTT_{ff}+HR	0.33 [0.25,0.39]	0.90 [0.89,0.92]	0.71 [0.67,0.75]	0.82 [0.79,0.85]
PTT_p+HR	0.06 [-0.01,0.14]	0.87 [0.85,0.89]	0.64 [0.59,0.69]	**0.82** [0.79,0.85]
PTT_D+HR	0.34 [0.28,0.40]	0.90 [0.88,0.91]	0.71 [0.67,0.75]	0.82 [0.79,0.85]
DAT+HR	0.09 [0.01,0.17]	0.86 [0.83,0.87]	0.56 [0.51,0.61]	0.81 [0.78,0.84]
PTT_{ff}+PTT_p	**0.55** [0.51,0.60]	0.83 [0.80,0.86]	0.66 [0.61,0.71]	0.68 [0.64,0.72]
PTT_{ff}+PTT_p+HR	0.71 [0.68,0.75]	0.91 [0.89,0.93]	0.71 [0.69,0.74]	**0.82** [0.80,0.85]
PTT_{ff}+PTT_D	0.34 [0.26,0.39]	0.72 [0.69,0.76]	0.66 [0.64,0.69]	0.51 [0.47,0.58]
PTT_{ff}+PTT_D+HR	0.34 [0.28,0.40]	0.90 [0.89,0.92]	**0.72** [0.68,0.76]	**0.83** [0.80,0.86]
PTT_{ff}+DAT	0.34 [0.26,0.39]	**0.91** [0.89,0.92]	**0.72** [0.68,0.75]	0.80 [0.77,0.83]
PTT_{ff}+DAT+HR	0.35 [0.29,0.43]	0.91 [0.89,0.92]	**0.72** [0.68,0.76]	0.82 [0.79,0.85]
PTT_{ff}+PTT_p+PTT_D	0.63 [0.60,0.66]	0.85 [0.83,0.88]	0.67 [0.66,0.71]	0.69 [0.64,0.74]
PTT_{ff}+PTT_p+PTT_D+HR	**0.72** [0.68,0.76]	**0.91** [0.89,0.93]	0.71 [0.70,0.75]	**0.83** [0.81,0.86]
PTT_{ff}+PTT_p+DAT	**0.72** [0.69,0.76]	**0.92** [0.90,0.93]	0.70 [0.64,0.73]	0.80 [0.76,0.84]
PTT_{ff}+PTT_p+DAT+HR	**0.72** [0.69,0.76]	**0.92** [0.91,0.94]	**0.72** [0.68,0.76]	0.82 [0.79,0.85]
PTT_{ff}+PTT_D+DAT	0.34 [0.27,0.40]	0.91 [0.90,0.93]	**0.72** [0.69,0.76]	0.81 [0.77,0.83]
PTT_{ff}+PTT_D+DAT+HR	0.35 [0.30,0.43]	0.91 [0.90,0.92]	0.71 [0.67,0.75]	**0.82** [0.80,0.86]
PTT_{ff}+PTT_p+PTT_D+DAT	**0.73** [0.69,0.77]	**0.92** [0.90,0.93]	0.72 [0.68,0.76]	0.81 [0.76,0.84]
PTT_{ff}+PTT_p+PTT_D+DAT+HR	**0.73** [0.71,0.77]	**0.92** [0.91,0.93]	0.72 [0.68,0.76]	**0.83** [0.80,0.86]

Table D: Accuracy of predictions using PTT measurements (the reference time is the aortic valve opening) from right femoral artery (RFem). Otherwise same caption as in Table A.

	aPWV	DBP	SBP	SV
HR	0.06 [-0.07,0.15]	**0.85** [0.83,0.87]	0.45 [0.38,0.51]	**0.81** [0.76,0.84]
PTT_{ff}	**0.75** [0.71,0.78]	0.35 [0.29,0.42]	0.59 [0.51,0.64]	0.08 [-0.02,0.15]
PTT_p	0.27 [0.18,0.34]	0.20 [0.11,0.27]	0.58 [0.53,0.63]	0.45 [0.38,0.51]
PTT_D	0.50 [0.43,0.56]	0.11 [0.01,0.20]	**0.63** [0.58,0.68]	0.24 [0.14,0.31]
DAT	0.09 [0.01,0.18]	0.77 [0.74,0.80]	0.56 [0.50,0.61]	0.79 [0.76,0.83]
PTT_{ff}+HR	0.75 [0.71,0.78]	0.92 [0.90,0.93]	0.75 [0.70,0.78]	0.82 [0.79,0.84]
PTT_p+HR	0.33 [0.27,0.40]	0.87 [0.85,0.89]	0.63 [0.58,0.68]	**0.82** [0.79,0.85]
PTT_D+HR	0.54 [0.48,0.59]	0.90 [0.88,0.92]	0.72 [0.68,0.76]	0.82 [0.79,0.84]
DAT+HR	0.52 [0.47,0.58]	0.87 [0.85,0.89]	0.64 [0.58,0.68]	0.81 [0.78,0.84]
PTT_{ff}+PTT_p	**0.80** [0.77,0.83]	0.67 [0.64,0.72]	0.65 [0.61,0.70]	0.52 [0.46,0.58]
PTT_{ff}+PTT_p+HR	0.82 [0.79,0.85]	**0.93** [0.91,0.94]	0.75 [0.71,0.78]	**0.82** [0.79,0.85]
PTT_{ff}+PTT_D	0.80 [0.76,0.83]	0.74 [0.71,0.78]	0.68 [0.67,0.71]	0.51 [0.49,0.56]
PTT_{ff}+PTT_D+HR	0.81 [0.77,0.83]	0.92 [0.91,0.93]	0.76 [0.73,0.79]	0.82 [0.79,0.84]
PTT_{ff}+DAT	0.75 [0.71,0.78]	**0.92** [0.91,0.94]	**0.75** [0.71,0.79]	0.80 [0.77,0.83]
PTT_{ff}+DAT+HR	0.75 [0.71,0.78]	0.93 [0.91,0.94]	0.75 [0.72,0.79]	0.81 [0.79,0.84]
PTT_{ff}+PTT_p+PTT_D	0.82 [0.80,0.85]	0.77 [0.77,0.78]	0.68 [0.66,0.71]	0.56 [0.55,0.61]
PTT_{ff}+PTT_p+PTT_D+HR	**0.83** [0.81,0.86]	**0.93** [0.91,0.94]	**0.76** [0.76,0.78]	**0.82** [0.79,0.85]
PTT_{ff}+PTT_p+DAT	**0.83** [0.80,0.86]	0.93 [0.91,0.94]	0.75 [0.73,0.78]	0.80 [0.76,0.84]
PTT_{ff}+PTT_p+DAT+HR	**0.83** [0.80,0.85]	**0.93** [0.91,0.94]	0.75 [0.73,0.78]	**0.82** [0.79,0.85]
PTT_{ff}+PTT_D+DAT	0.81 [0.78,0.84]	0.92 [0.91,0.94]	**0.76** [0.74,0.79]	0.80 [0.76,0.83]
PTT_{ff}+PTT_D+DAT+HR	0.81 [0.77,0.84]	**0.93** [0.92,0.94]	**0.76** [0.75,0.79]	0.81 [0.79,0.85]
PTT_{ff}+PTT_p+PTT_D+DAT	**0.84** [0.82,0.88]	0.93 [0.91,0.94]	**0.77** [0.76,0.79]	0.80 [0.78,0.84]
PTT_{ff}+PTT_p+PTT_D+DAT+HR	**0.84** [0.82,0.86]	**0.93** [0.92,0.94]	**0.77** [0.76,0.79]	**0.82** [0.80,0.85]

Table E: Accuracy of predictions using PAT measurements (all timings calculated as a difference to the ignition of the pulse in E_{fw} for LV) from left carotid artery (LCA). The predictions with largest Pearson correlations are highlighted: one signal (red), two signals (blue) and five most accurate combination (black). Signals: heart rate (HR) and pulse transit arrival to the foot of signal ($\mathrm{PAT_{ff}}$), peak of signal ($\mathrm{PAT_p}$), the point of steepest raise (PAT_D), and the dicrotic notch (DAT).

	aPWV	DBP	SBP	SV
HR	0.06 [-0.08,0.14]	**0.85** [0.83,0.87]	0.46 [0.41,0.52]	**0.81** [0.77,0.84]
$\mathrm{PAT_{ff}}$	**0.52** [0.47,0.57]	0.19 [0.09,0.27]	**0.62** [0.57,0.67]	0.18 [0.10,0.25]
$\mathrm{PAT_p}$	0.25 [0.19,0.33]	0.67 [0.64,0.71]	0.54 [0.46,0.61]	0.54 [0.49,0.59]
PAT_D	0.47 [0.41,0.52]	0.05 [-0.03,0.13]	0.61 [0.52,0.66]	0.24 [0.09,0.31]
DAT	-0.03 [-0.15,0.04]	0.83 [0.80,0.85]	0.48 [0.43,0.55]	0.79 [0.75,0.82]
$\mathrm{PAT_{ff}}$+HR	0.55 [0.51,0.60]	0.93 [0.92,0.94]	**0.71** [0.67,0.76]	0.81 [0.78,0.84]
$\mathrm{PAT_p}$+HR	0.28 [0.21,0.37]	0.90 [0.88,0.91]	0.67 [0.63,0.72]	**0.82** [0.79,0.85]
PAT_D+HR	0.52 [0.47,0.57]	0.92 [0.90,0.93]	0.68 [0.64,0.73]	0.81 [0.78,0.84]
DAT+HR	0.02 [-0.06,0.05]	0.85 [0.83,0.87]	0.48 [0.30,0.52]	0.81 [0.78,0.84]
$\mathrm{PAT_{ff}}$+$\mathrm{PAT_p}$	**0.72** [0.69,0.76]	0.81 [0.79,0.83]	0.68 [0.64,0.73]	0.61 [0.57,0.67]
$\mathrm{PAT_{ff}}$+$\mathrm{PAT_p}$+HR	**0.78** [0.75,0.81]	0.94 [0.92,0.95]	**0.75** [0.71,0.79]	**0.82** [0.80,0.85]
$\mathrm{PAT_{ff}}$+PAT_D	0.58 [0.56,0.63]	0.79 [0.76,0.83]	0.66 [0.65,0.68]	0.53 [0.49,0.59]
$\mathrm{PAT_{ff}}$+PAT_D+HR	0.63 [0.63,0.64]	0.95 [0.93,0.96]	0.73 [0.70,0.78]	**0.82** [0.79,0.85]
$\mathrm{PAT_{ff}}$+DAT	0.56 [0.52,0.62]	**0.95** [0.93,0.96]	0.71 [0.67,0.75]	0.79 [0.76,0.82]
$\mathrm{PAT_{ff}}$+DAT+HR	0.56 [0.52,0.62]	0.95 [0.93,0.96]	0.71 [0.68,0.76]	0.81 [0.79,0.84]
$\mathrm{PAT_{ff}}$+$\mathrm{PAT_p}$+PAT_D	0.76 [0.75,0.79]	0.89 [0.88,0.90]	0.70 [0.70,0.71]	0.68 [0.67,0.68]
$\mathrm{PAT_{ff}}$+$\mathrm{PAT_p}$+PAT_D+HR	**0.79** [0.77,0.82]	**0.95** [0.95,0.96]	**0.76** [0.72,0.80]	**0.82** [0.80,0.85]
$\mathrm{PAT_{ff}}$+$\mathrm{PAT_p}$+DAT	0.76 [0.73,0.79]	0.95 [0.94,0.96]	0.75 [0.73,0.79]	0.80 [0.75,0.84]
$\mathrm{PAT_{ff}}$+$\mathrm{PAT_p}$+DAT+HR	**0.78** [0.75,0.82]	0.95 [0.94,0.96]	**0.75** [0.73,0.79]	**0.82** [0.79,0.85]
$\mathrm{PAT_{ff}}$+PAT_D+DAT	0.65 [0.62,0.63]	**0.96** [0.94,0.97]	0.72 [0.69,0.77]	0.79 [0.75,0.83]
$\mathrm{PAT_{ff}}$+PAT_D+DAT+HR	0.65 [0.63,0.64]	**0.96** [0.94,0.97]	0.73 [0.71,0.77]	0.81 [0.78,0.84]
$\mathrm{PAT_{ff}}$+$\mathrm{PAT_p}$+PAT_D+DAT	**0.78** [0.71,0.81]	**0.96** [0.95,0.97]	**0.76** [0.74,0.80]	0.80 [0.76,0.85]
$\mathrm{PAT_{ff}}$+$\mathrm{PAT_p}$+PAT_D+DAT+HR	**0.79** [0.76,0.82]	**0.96** [0.95,0.97]	**0.76** [0.75,0.79]	0.80 [0.77,0.82]

Table F: Accuracy of predictions using difference of PTT measurements from right femoral artery and left carotid artery (LCA - Fem). Otherwise same caption as in Table A.

	aPWV	DBP	SBP	SV
HR	0.06 [-0.07,0.13]	**0.85** [0.83,0.87]	0.46 [0.40,0.51]	**0.81** [0.77,0.84]
$\mathrm{PTT_{ff}}$	**0.76** [0.72,0.79]	0.35 [0.28,0.42]	0.59 [0.53,0.64]	0.10 [0.02,0.17]
$\mathrm{PTT_p}$	0.42 [0.36,0.48]	0.67 [0.63,0.71]	0.32 [0.26,0.38]	0.49 [0.43,0.55]
PTT_D	0.48 [0.39,0.54]	0.17 [0.09,0.24]	**0.60** [0.51,0.66]	0.21 [0.10,0.30]
DAT	0.75 [0.72,0.78]	0.36 [0.28,0.44]	0.59 [0.52,0.64]	0.10 [0.00,0.19]
$\mathrm{PTT_{ff}}$+HR	0.76 [0.73,0.79]	0.92 [0.91,0.93]	0.75 [0.70,0.78]	0.82 [0.79,0.84]
$\mathrm{PTT_p}$+HR	0.45 [0.39,0.51]	0.88 [0.86,0.90]	0.62 [0.58,0.67]	**0.83** [0.80,0.85]
PTT_D+HR	0.52 [0.46,0.58]	0.90 [0.88,0.91]	0.70 [0.66,0.75]	0.82 [0.79,0.85]
DAT+HR	0.76 [0.72,0.79]	**0.92** [0.91,0.94]	**0.75** [0.70,0.78]	0.82 [0.79,0.84]
$\mathrm{PTT_{ff}}$+$\mathrm{PTT_p}$	0.80 [0.77,0.83]	0.71 [0.67,0.74]	0.63 [0.58,0.68]	0.52 [0.46,0.57]
$\mathrm{PTT_{ff}}$+$\mathrm{PTT_p}$+HR	0.82 [0.79,0.85]	**0.93** [0.91,0.94]	0.75 [0.72,0.79]	**0.82** [0.80,0.86]
$\mathrm{PTT_{ff}}$+PTT_D	**0.83** [0.81,0.87]	0.44 [0.40,0.50]	0.64 [0.61,0.68]	0.24 [0.16,0.31]
$\mathrm{PTT_{ff}}$+PTT_D+HR	0.83 [0.81,0.86]	0.92 [0.90,0.93]	**0.76** [0.73,0.79]	0.82 [0.79,0.84]
$\mathrm{PTT_{ff}}$+DAT	0.76 [0.72,0.79]	0.36 [0.30,0.44]	0.59 [0.55,0.64]	0.10 [0.02,0.18]
$\mathrm{PTT_{ff}}$+DAT+HR	0.76 [0.73,0.81]	0.92 [0.91,0.94]	0.75 [0.71,0.78]	0.82 [0.79,0.84]
$\mathrm{PTT_{ff}}$+$\mathrm{PTT_p}$+PTT_D	**0.86** [0.84,0.88]	0.73 [0.69,0.76]	0.68 [0.65,0.73]	0.54 [0.48,0.60]
$\mathrm{PTT_{ff}}$+$\mathrm{PTT_p}$+PTT_D+HR	**0.87** [0.83,0.89]	**0.93** [0.90,0.94]	**0.77** [0.77,0.78]	**0.82** [0.79,0.85]
$\mathrm{PTT_{ff}}$+$\mathrm{PTT_p}$+DAT	0.80 [0.76,0.83]	0.74 [0.71,0.78]	0.65 [0.62,0.70]	0.61 [0.54,0.66]
$\mathrm{PTT_{ff}}$+$\mathrm{PTT_p}$+DAT+HR	0.83 [0.78,0.86]	**0.93** [0.91,0.94]	**0.75** [0.72,0.79]	**0.82** [0.80,0.85]
$\mathrm{PTT_{ff}}$+PTT_D+DAT	0.83 [0.81,0.87]	0.44 [0.40,0.53]	0.64 [0.61,0.68]	0.24 [0.18,0.32]
$\mathrm{PTT_{ff}}$+PTT_D+DAT+HR	**0.84** [0.82,0.85]	0.92 [0.90,0.94]	**0.76** [0.74,0.79]	0.81 [0.78,0.84]
$\mathrm{PTT_{ff}}$+$\mathrm{PTT_p}$+PTT_D+DAT	**0.86** [0.84,0.88]	0.76 [0.71,0.79]	0.70 [0.71,0.73]	0.63 [0.46,0.69]
$\mathrm{PTT_{ff}}$+$\mathrm{PTT_p}$+PTT_D+DAT+HR	**0.87** [0.85,0.89]	**0.93** [0.91,0.94]	**0.76** [0.77,0.79]	**0.82** [0.79,0.85]

Table G: Accuracy of predictions using difference of PTT measurements from left and right carotid artery (LCA-RCA). Otherwise same caption as in Table A.

	aPWV	DBP	SBP	SV
HR	0.06 [-0.08,0.14]	**0.85** [0.83,0.87]	**0.46** [0.39,0.51]	**0.81** [0.76,0.84]
PTT$_{\mathrm{ff}}$	**0.75** [0.72,0.78]	0.35 [0.30,0.43]	0.40 [0.34,0.45]	0.01 [-0.06,0.08]
PTT$_{\mathrm{p}}$	0.24 [0.18,0.31]	0.46 [0.40,0.52]	0.22 [0.16,0.31]	0.29 [0.23,0.35]
PTT$_D$	0.59 [0.54,0.64]	0.31 [0.24,0.37]	0.35 [0.29,0.41]	0.07 [0.00,0.16]
DAT	0.66 [0.47,0.72]	0.17 [0.06,0.25]	0.21 [0.10,0.38]	0.12 [-0.03,0.21]
PTT$_{\mathrm{ff}}$+HR	0.75 [0.71,0.78]	**0.89** [0.88,0.91]	**0.63** [0.57,0.67]	0.81 [0.79,0.85]
PTT$_{\mathrm{p}}$+HR	0.31 [0.24,0.42]	0.87 [0.85,0.89]	0.57 [0.52,0.63]	**0.82** [0.80,0.85]
PTT$_D$+HR	0.60 [0.55,0.64]	0.88 [0.86,0.90]	0.60 [0.54,0.65]	0.82 [0.79,0.85]
DAT+HR	0.61 [0.31,0.69]	0.88 [0.85,0.90]	0.46 [0.28,0.61]	0.81 [0.77,0.84]
PTT$_{\mathrm{ff}}$+PTT$_{\mathrm{p}}$	**0.75** [0.72,0.79]	0.56 [0.50,0.61]	0.44 [0.38,0.50]	0.30 [0.24,0.37]
PTT$_{\mathrm{ff}}$+PTT$_{\mathrm{p}}$+HR	0.75 [0.73,0.79]	**0.90** [0.88,0.92]	**0.68** [0.66,0.72]	**0.82** [0.80,0.85]
PTT$_{\mathrm{ff}}$+PTT$_D$	0.75 [0.72,0.78]	0.52 [0.48,0.58]	0.45 [0.40,0.51]	0.38 [0.33,0.45]
PTT$_{\mathrm{ff}}$+PTT$_D$+HR	0.75 [0.72,0.79]	0.89 [0.88,0.91]	0.63 [0.58,0.69]	0.82 [0.80,0.85]
PTT$_{\mathrm{ff}}$+DAT	0.74 [0.69,0.77]	0.35 [0.05,0.61]	0.44 [0.39,0.49]	0.49 [0.41,0.57]
PTT$_{\mathrm{ff}}$+DAT+HR	0.73 [0.68,0.76]	**0.90** [0.88,0.91]	0.63 [0.59,0.69]	0.81 [0.78,0.84]
PTT$_{\mathrm{ff}}$+PTT$_{\mathrm{p}}$+PTT$_D$	**0.76** [0.73,0.80]	0.58 [0.35,0.63]	0.44 [0.40,0.52]	0.31 [-0.13,0.42]
PTT$_{\mathrm{ff}}$+PTT$_{\mathrm{p}}$+PTT$_D$+HR	**0.76** [0.75,0.78]	**0.90** [0.89,0.92]	**0.69** [0.69,0.70]	**0.82** [0.80,0.85]
PTT$_{\mathrm{ff}}$+PTT$_{\mathrm{p}}$+DAT	**0.75** [0.73,0.79]	0.65 [0.51,0.69]	0.44 [0.38,0.52]	0.39 [-0.01,0.53]
PTT$_{\mathrm{ff}}$+PTT$_{\mathrm{p}}$+DAT+HR	0.74 [0.71,0.78]	**0.90** [0.89,0.92]	**0.68** [0.67,0.72]	**0.82** [0.80,0.85]
PTT$_{\mathrm{ff}}$+PTT$_D$+DAT	0.75 [0.70,0.78]	0.66 [0.46,0.73]	0.35 [0.25,0.43]	0.38 [0.07,0.55]
PTT$_{\mathrm{ff}}$+PTT$_D$+DAT+HR	0.74 [0.70,0.78]	0.89 [0.88,0.91]	**0.63** [0.60,0.69]	0.82 [0.79,0.85]
PTT$_{\mathrm{ff}}$+PTT$_{\mathrm{p}}$+PTT$_D$+DAT	**0.76** [0.74,0.80]	0.68 [0.63,0.73]	0.43 [0.42,0.50]	0.48 [0.25,0.55]
PTT$_{\mathrm{ff}}$+PTT$_{\mathrm{p}}$+PTT$_D$+DAT+HR	**0.76** [0.75,0.77]	**0.90** [0.90,0.91]	**0.69** [0.69,0.70]	**0.83** [0.82,0.85]

Table H: Accuracy of predictions using difference of PTT measurements from left and right radialis artery (LRad-RRad). Otherwise same caption as in Table A.

	aPWV	DBP	SBP	SV
HR	0.06 [-0.08,0.13]	**0.85** [0.83,0.87]	**0.46** [0.40,0.52]	**0.81** [0.77,0.84]
PTT$_{\mathrm{ff}}$	**0.67** [0.63,0.71]	0.19 [0.13,0.26]	0.22 [0.16,0.30]	0.03 [-0.04,0.14]
PTT$_{\mathrm{p}}$	0.46 [0.41,0.51]	0.59 [0.53,0.63]	0.29 [0.23,0.36]	0.45 [0.40,0.50]
PTT$_D$	0.65 [0.60,0.69]	0.19 [0.14,0.29]	0.22 [0.16,0.29]	0.02 [-0.05,0.10]
DAT	0.62 [0.58,0.67]	0.12 [0.01,0.18]	0.25 [0.18,0.32]	0.17 [0.12,0.26]
PTT$_{\mathrm{ff}}$+HR	0.67 [0.63,0.71]	0.87 [0.85,0.89]	0.51 [0.46,0.57]	0.81 [0.78,0.84]
PTT$_{\mathrm{p}}$+HR	0.49 [0.44,0.55]	**0.88** [0.86,0.90]	**0.53** [0.47,0.59]	0.81 [0.78,0.84]
PTT$_D$+HR	0.65 [0.60,0.69]	0.87 [0.85,0.89]	0.51 [0.46,0.57]	0.81 [0.78,0.84]
DAT+HR	0.64 [0.60,0.69]	0.87 [0.85,0.89]	0.52 [0.47,0.57]	**0.81** [0.79,0.84]
PTT$_{\mathrm{ff}}$+PTT$_{\mathrm{p}}$	**0.76** [0.73,0.79]	0.62 [0.57,0.68]	0.34 [0.28,0.41]	0.47 [0.42,0.53]
PTT$_{\mathrm{ff}}$+PTT$_{\mathrm{p}}$+HR	**0.77** [0.74,0.80]	**0.89** [0.86,0.91]	**0.55** [0.49,0.61]	0.81 [0.79,0.85]
PTT$_{\mathrm{ff}}$+PTT$_D$	0.68 [0.62,0.72]	0.38 [0.34,0.46]	0.30 [0.25,0.38]	0.02 [-0.05,0.02]
PTT$_{\mathrm{ff}}$+PTT$_D$+HR	0.68 [0.64,0.74]	0.87 [0.85,0.89]	0.51 [0.46,0.58]	0.81 [0.78,0.84]
PTT$_{\mathrm{ff}}$+DAT	0.67 [0.63,0.71]	0.44 [0.43,0.48]	0.24 [0.18,0.34]	0.36 [0.27,0.46]
PTT$_{\mathrm{ff}}$+DAT+HR	0.67 [0.65,0.71]	0.87 [0.86,0.89]	0.55 [0.54,0.58]	**0.81** [0.79,0.84]
PTT$_{\mathrm{ff}}$+PTT$_{\mathrm{p}}$+PTT$_D$	0.77 [0.71,0.80]	0.63 [0.61,0.67]	0.34 [0.23,0.42]	0.47 [0.30,0.53]
PTT$_{\mathrm{ff}}$+PTT$_{\mathrm{p}}$+PTT$_D$+HR	**0.78** [0.68,0.81]	**0.89** [0.87,0.91]	**0.55** [0.51,0.61]	**0.81** [0.79,0.84]
PTT$_{\mathrm{ff}}$+PTT$_{\mathrm{p}}$+DAT	0.76 [0.73,0.80]	0.69 [0.68,0.73]	0.37 [0.28,0.44]	0.52 [0.27,0.60]
PTT$_{\mathrm{ff}}$+PTT$_{\mathrm{p}}$+DAT+HR	**0.77** [0.70,0.81]	**0.89** [0.88,0.91]	**0.58** [0.59,0.62]	**0.81** [0.79,0.85]
PTT$_{\mathrm{ff}}$+PTT$_D$+DAT	0.68 [0.63,0.74]	0.47 [0.44,0.53]	0.26 [0.21,0.35]	0.37 [0.32,0.45]
PTT$_{\mathrm{ff}}$+PTT$_D$+DAT+HR	0.68 [0.68,0.70]	0.88 [0.87,0.89]	**0.56** [0.58,0.59]	0.81 [0.78,0.84]
PTT$_{\mathrm{ff}}$+PTT$_{\mathrm{p}}$+PTT$_D$+DAT	**0.77** [0.66,0.80]	0.67 [0.66,0.71]	0.36 [0.23,0.43]	0.57 [0.48,0.61]
PTT$_{\mathrm{ff}}$+PTT$_{\mathrm{p}}$+PTT$_D$+DAT+HR	**0.78** [0.75,0.81]	**0.89** [0.88,0.90]	**0.58** [0.60,0.61]	**0.81** [0.79,0.85]
